# A ketogenic diet alters mTOR activity, systemic metabolism and potentially prevents collagen degradation associated with chronic alcohol consumption in mice

**DOI:** 10.1007/s11306-023-02006-w

**Published:** 2023-04-19

**Authors:** Luciano Willemse, Karin Terburgh, Roan Louw

**Affiliations:** Human Metabolomics, Faculty of Natural and Agricultural Sciences, North-West University (Potchefstroom Campus), Private Bag X6001, Potchefstroom, South Africa

**Keywords:** Ketogenic diet, Mtor, Metabolomics, Redox, Western blot, Metabolic reprogramming

## Abstract

**Introduction:**

A ketogenic diet (KD), which is a high fat, low carbohydrate diet has been shown to inhibit the mammalian target of rapamycin (mTOR) pathway and alter the redox state. Inhibition of the mTOR complex has been associated with the attenuation and alleviation of various metabolic and- inflammatory diseases such as neurodegeneration, diabetes, and metabolic syndrome. Various metabolic pathways and signalling mechanisms have been explored to assess the therapeutic potential of mTOR inhibition. However, chronic alcohol consumption has also been reported to alter mTOR activity, the cellular redox- and inflammatory state. Thus, a relevant question that remains is what effect chronic alcohol consumption would have on mTOR activity and overall metabolism during a KD-based intervention.

**Objectives:**

The aim of this study was to evaluate the effect of alcohol and a KD on the phosphorylation of the mTORC1 target p70S6K, systemic metabolism as well as the redox- and inflammatory state in a mouse model.

**Methods:**

Mice were fed either a control diet with/without alcohol or a KD with/without alcohol for three weeks. After the dietary intervention, samples were collected and subjected towards western blot analysis, multi-platform metabolomics analysis and flow cytometry.

**Results:**

Mice fed a KD exhibited significant mTOR inhibition and reduction in growth rate. Alcohol consumption alone did not markedly alter mTOR activity or growth rate but moderately increased mTOR inhibition in mice fed a KD. In addition, metabolic profiling showed alteration of several metabolic pathways as well as the redox state following consumption of a KD and alcohol. A KD was also observed to potentially prevent bone loss and collagen degradation associated with chronic alcohol consumption, as indicated by hydroxyproline metabolism.

**Conclusion:**

This study sheds light on the influence that a KD alongside alcohol intake can exert on not just mTOR, but also their effect on metabolic reprogramming and the redox state.

**Supplementary Information:**

The online version contains supplementary material available at 10.1007/s11306-023-02006-w.

## Introduction

A ketogenic diet (KD), defined as a high fat, low carbohydrate diet, has been shown to attenuate various disorders that affect brain function and neurological metabolism, as well as refractory epilepsy (Vinnig, 1999), mitochondrial disease (Sperl, 1997), diabetes (Qu et., 2021) and glucose transporter defects (Bolla et al., 2019). More recently, the KD has been one of the most significantly researched approaches with regards to weight loss and as a strategy to manage obesity (Paoli, 2014). Several mechanisms have been assessed regarding the potential therapeutic effects of a KD, including, but not limited to, ketosis and ketone bodies (Freeman et al., 2010), inflammatory pathways (Dupuis et al., 2015), oxidative stress regulation (Jin et al., 2014) and the mammalian target of rapamycin (mTOR) pathway (McDaniel et al., 2011). Of these potential mechanisms, the disease ameliorating effects of the mTOR signalling pathway has been of particular interest, resulting in extensive research thereof (Genzer et al., 2016). The mTOR pathway is considered a central control hub for growth and metabolism (Wullschleger et al., 2006), activated by phosphoinositide-3 kinase (PI3K)/ protein kinase B (Akt) signalling during conditions of ample nutrient and growth factor availability, and inhibited by AMP-activated protein kinase (AMPK) during energy deficiency (Jewell and Guan, 2013). mTOR is a protein kinase that integrates energy, nutrient, and growth signals to modulate several cellular processes, and exists in two distinct functional complexes namely mTOR complex 1 (mTORC1) and mTOR complex 2 (mTORC2). The former is considered the primary regulatory complex with regard to cellular homeostasis, stress responses, autophagy, and energy metabolism (Laplante & Sabatini, 2013; Morita et al., 2013, 2015), and also plays a particularly significant role in managing the Krebs cycle (TCA cycle), mitochondrial biogenesis, as well as oxidative stress (Morita et al., 2015; Zhao et al., 2017). As such, mTORC1 has been a target of interest in the development of therapeutic intervention strategies for several disorders such as epilepsy, cancer, neurodegeneration, diabetes, and mitochondrial diseases (Hay & Sonenberg, 2004; Johnson et al., 2013; McDaniel et al., 2011; Xie et al., 2016). When the AMPK or Akt pathway is stimulated by growth factors, nutrients, or insulin signalling, it leads to mTORC1 activation and phosphorylation of the downstream protein, p70S6K, to regulate protein synthesis, cell growth and proliferation (Hay & Sonenberg, 2004). The phosphorylation of p70S6K is one of the best characterised downstream effects of mTOR, as such, the measurement of the activity of this protein makes it a suitable target for monitoring mTOR inhibition (Hay & Sonenberg, 2004). Inhibition of mTORC1 ultimately leads to the alleviation of several of these diseases, thereby implicating distinct metabolic pathways and signalling cascades. Accordingly, administration of the potent mTOR inhibitor, rapamycin, has been reported to prevent epilepsy, disrupt tumour metabolism as well as impair mitochondrial disease progression in various animal models (Johnson et al., 2013; Xie et al., 2016; Zeng et al., 2008). Various studies have also evaluated the inhibitory effect of a KD on mTOR activity in rodent models of mitochondrial disease, epilepsy, and longevity (McDaniel et al., 2011; Roberts et al., 2017; Singh et al., 2018). Together, these findings suggest a credible relationship between a KD and mTOR activity. Additionally, alcohol consumption is known to affect ketone body metabolism, alter the cellular redox state, and impair mTOR phosphorylation (Feldstein & Bailey, 2011; Lang et al., 2003; Veech et al., 1972; Zakhari, 2006). Numerous studies have also evaluated the effect of a KD on alcohol withdrawal symptoms as well as alcohol addiction in both rodents and humans (Blanco-Gandía et al., 2021; Bornebusch et al., 2021; Dencker et al., 2018; Wiers et al., 2021). In these studies researchers observed that a KD decreases alcohol consumption, as well as alleviating alcohol withdrawal symptoms. Moreover, an increase in worldwide alcohol consumption has been observed, where alcohol use disorder (AUD) has been linked to around 5% of global deaths (Rehm & Imtiaz, 2016). Considering this, the effect of chronic alcohol consumption on the mTOR axis and overall metabolism during a KD-based intervention for weight management and disease treatment comes into question. To address this concept, the current study aimed to first investigate the isolated and combined effects of chronic alcohol consumption and a KD on the mTORC1 phosphorylation state, systemic metabolism, and redox state in a healthy mouse model. For this study the mice consuming the normal diet along with alcohol consumption served as a control for NADH-reductive stress, a redox state shift towards a more decreased NAD^+^/NADH ratio. This was used to evaluate whether a ketogenic diet could prevent or even reverse this shift in the redox state. Following the dietary intervention, mouse liver tissue was used to assess p70S6K1, a downstream protein of mTOR phosphorylation, whereas multiplatform-based metabolomics analyses of mouse urinary samples were utilised to evaluate systemic metabolic and redox fluctuations within the various dietary groups.

## Methods and materials

Complete details regarding the methods and materials used can be found in the Supplementary material.

### Animals, dietary intervention and sampling

Equal numbers of male mice, heterozygous (HET) or wild-type (WT) for *Ndufs4*^*tm1.1Rpa*^ (Fig. S2), were used interchangeably in this study since both genotypes can serve as healthy controls (Kruse et al., 2008), due to there being no difference in phenotype between the two genotypes (van de Wal et al., 2022). All four treatment groups contained equal numbers of WT and heterozygote animals, while the metabolomics data showed no natural separation between the WT and heterozygote animals (Fig. S2). Original *Ndufs4* HET breeding pairs (B6.129S4-*Ndufs4*^*tm1.1Rpa*^/J) were acquired from Jackson Laboratory (JAX; ME, USA, stock #027,058). Only male mice were used in this study due to the known differences in hormone cycles, tissue-specific metabolism as well as metabolic profiles between sexes (Ruoppolo et al., 2018; Terburgh et al., 2021). Standard polymerase chain reaction genotyping was used as described previously (Valsecchi et al., 2010) to distinguish *Ndufs4* mouse genotypes and confirm that mice of the correct genotype were used for the study. Mice were housed and bred at the specific pathogen-free (SPF) unit of the Vivarium (SAVC reg. #FR15/13458) of the Preclinical Drug Development Platform (PCDDP, NWU) under the following conditions: a day/night cycle of 12 h; temperature of 22 °C ± 2 °C; and relative humidity of 55% ± 10%. After postnatal day (P) 23, a total of 40 mice were randomly assigned to one of four dietary intervention groups (n = 10 animals per group), with ad libitum access to either; i) a control diet, referred to as a normal diet (ND) in this study (standard laboratory chow, RB2005, LabChef, NutritionHub) and drinking water; ii) ND with 5% (v/v) alcohol in drinking water for the first week and 10% (v/v) alcohol in drinking water for the remainder of the study; iii) a KD and drinking water; or iv) a KD with 5% (v/v) alcohol in drinking water for the first week and 10% (v/v) alcohol in drinking water for the remainder of the study. The ND contained (% of total kcal) 20% protein, 75% carbohydrate, and 5% fat; whereas the KD contained 8.2% protein, 91.7% fat, and < 0.1% carbohydrate. Detailed information regarding the ingredient composition of the KD can be found in the Supplementary material (Table S1). These dietary interventions lasted approximately three weeks, with mice weighed three times a week (around the same time intervals) to assess growth rates. Animals were euthanised on postnatal day P46-47, after completing the three-week dietary intervention. Mice were placed in metabolic cages the day before euthanasia to collect fasting urinary samples. Mice were euthanised via exsanguination as described by the American Veterinary Medical Association (AVMA) (AVMA, 2020) by a research animal technician (PCDDP, NWU, RSA) after a 16–18 h fasting period at the same time of day (10:00 to 11:00 AM) to reduce inter-animal physiological variation because of the differences in nocturnal feeding habits as well as that of the stages of the circadian cycle (Griffin et al., 2006). Finally, after blood and liver tissues were collected, all samples were swiftly snap-frozen in liquid nitrogen to quench metabolic activity, and subsequently stored at − 80 °C.

The AnimCare animal research ethics committee of North-West University approved (NWU-00516-20-A5) the animal protocols used in this study. All animals were maintained, and all procedures performed, in accordance with the code of ethics in research, training, and testing of drugs in South Africa and complied with national legislation.

### SDS-PAGE and western blotting of liver pS6K1

Liver tissue (~ 100 mg) was homogenised in HEPES lysis buffer (~ 1 ml) with a Glass/Teflon® Tight Fitting Potter–Elvehjem Homogeniser (Glas-Col) using 15 strokes. Thereafter, liver homogenates were centrifuged with a Heraeus™ Multifuge™ X3 centrifuge (Thermo Fisher Scientific). Subsequently, supernatants were collected, and protein concentrations were determined using the BCA assay. Supernatants were kept on ice throughout the procedure, which were subsequently vortexed, and centrifuged at 1000 xg before heat denaturation for five min at 100 °C. For each dietary group, aliquots (20 μg) of the protein were separated via a sodium dodecyl sulfate–polyacrylamide gel (SDS-PAGE), run at room temperature. Afterwards, the gel was transferred to nitrocellulose and blocked. After that, the membranes were incubated with phospho-p70S6 kinase primary antibodies (Cell Signaling Technologies) at 4 °C overnight. Next, the membranes were incubated with secondary antibodies at room temperature. Lastly, chemiluminescent detection was achieved using a Western ECL substrate (Bio-Rad, Johannesburg, RSA) and a ChemiDoc™ MP imaging system (Bio-Rad). Finally, the results were analysed via Image Lab™ v. 6.1 software (Bio-Rad,) in order attain the density ratio of the target proteins relative to β-actin, used as the control protein. Detailed information regarding the procedures and antibody dilutions are described in the supplementary materials.

### Determination of urinary creatinine concentration

The creatinine concentration of each urinary sample was spectrophotometrically quantified using the QuantiChrom™ Creatinine Assay Kit (BioAssay Systems). In this assay, creatinine reacts with picric acid in an alkaline solution forming a yellow orange adduct. The increase in absorbance of this adduct is measured at a wavelength of 510 nm. For the assay, 10 μl of mouse urine was mixed with 200 μl working solution (0.1 M sodium hydroxide (NaOH); 0.3 mM ethylenediaminetetraacetic acid (EDTA); 0.1% (v/v) DMSO; 0.0004% (v/v) Tween®20; and 3 mM picric acid), in duplicate, in a 96-well plate. The samples were then analysed via a Synergy™ HT Multi-detection microplate reader (Biotek® Instruments) by assessing the increase in linear trend of absorbance at 510 nm for 5 min, in 1 min intervals. Absolute quantities were determined by making use of a standard curve, ranging from 0 to 50 mg%.

### Metabolomics sample preparation

For all the metabolomics analyses, a pre-determined volume (~ 20–250 μl) of urine containing 0.0625 μmole creatinine was added to microcentrifuge tubes, with the addition of internal standards (ISs). Urine samples were analysed via untargeted (gas chromatography time-of-flight mass spectrometry (GC-TOF-MS) and nuclear magnetic resonance (^1^H-NMR) spectroscopy) and targeted [liquid chromatography-tandem mass spectrometry (LC–MS/MS)] techniques. Due to the small volumes of some of the KD + Alc. urine samples obtained, only 6 biological replicates could be used for this group. For GC-TOF-MS analyses, 16.14 μl IS mixture, which contained 3-phenylbutyrate (100 ppm), norleucine (100 ppm) and *N,N*-dimethylphenylalanine (50 ppm), was added per sample. For LC–MS/MS analyses, 25 μl IS mixture was added. This mixture consisted of *N,N*-dimethylphenylalanine, ring-^2^H_5_-phenylalanine, ^2^H_10_-isoleucine, ^2^H_8_-valine, L-Carnitine-(methyl-*d*_*3*_), ^2^H_3_-octanoylcarnitine and ^2^H_3_-octadecanoylcarnitine, each with a final concentration of 2.5 ppm. After the addition of the IS mixture, mouse urine samples were deproteinised by adding three volumes of ice-cold methanol. The samples were then vortexed, incubated for 20 min at -20 °C, centrifuged at 15 000 xg for 10 min and finally dried under a gentle stream of nitrogen gas at 37 °C. Additionally, another 80 μl of methanol was added to all dried samples and then dried again to guarantee that the samples were completely dry and all water removed prior to derivatisation. For ^1^H-NMR analyses, urine samples did not undergo deproteinisation as samples were dried and then resuspended in sterile, filtered water and centrifuged to remove macromolecules and other insoluble particulates prior to ^1^H-NMR analyses. Also, ISs such as 3-trimethylsilyl propionic acid (TSP) were added during NMR sample preparation. LC–MS samples were butylated, while GC–MS samples were oximated and silyated prior to analysis. Additional details regarding sample preparation are described in the Supplementary material.

### LC–MS/MS analyses

For targeted LC–MS/MS analyses, urinary samples were analysed using a 1200 series liquid chromatograph coupled to a 6410-triple quadrupole-mass spectrometer (Agilent Technologies) as previously described (Terburgh et al., 2019). The chromatographic separation was accomplished by injecting samples (1 μl) on a C18 Zorbax SB-Aq (150 mm × 2.1 mm × 1.8 μm) reverse phase column (Agilent Technologies), kept at 35 °C, with a flow rate of 0.2 ml/min during the entire run. Furthermore, the mobile phases used comprised of H_2_O (solvent A) and acetonitrile (solvent B). Both mobile phases were acidified with 0.1% formic acid to enable protonation of metabolites (Cech & Enke, 2001). The chromatographic gradient started at a 5% mobile phase B and was maintained for 1 min, it was then followed with an increase to 23% of mobile phase B over a duration of 7 min. In addition, an isocratic hold of around 4 min was maintained at a 23% mobile phase B. Subsequently, mobile phase B was increased to 100% at 15 min and was kept there for 5 min. Lastly, the gradient declined to a 5% mobile phase B after 1 min and was maintained for 7 min (post-run) to guarantee that the column is in equilibrium before proceeding to the next analysis. The effluent coming from the liquid chromatograph was then diverted to the mass spectrometer, which made use of the positive electrospray ionisation (ESI) mode using the following source parameters: capillary voltage of 3 500 V; nitrogen drying gas at a flow rate of 7.5 l/min at 300 °C; and nebuliser pressure of 30 psi. The spectra were acquired via the multiple reaction monitoring (MRM) mode with an electron multiplier voltage (EMV) of 300 V and a dwell time of 45 ms for all compounds, with the MRM parameters set up for specific amino acids, -derivatives, acylcarnitines, and isotopes (Table S2).

### GC-TOF–MS analyses

For untargeted GC-TOF–MS analyses, samples were analysed via a 7890A gas chromatograph (Agilent Technologies) joined to a Pegaus HT time-of-flight mass spectrometer (Leco). Samples (1 μl) were injected using a split ratio of 1:10. The temperature of the inlet was 250 °C throughout the sample injection. Compounds were chromatographically separated using a DB-5 MS capillary column (30 m × 250 μm × 0.25 μm) (Restek), and helium was used as a carrier gas at a constant flow rate of 1.5 ml/min. For mass spectrometry, the separated compounds were subjected to electron impact ionisation (EI) (70 eV), with the ion source temperature set at 200 °C. After a solvent delay of 250 s, mass spectra were acquired (50–950 m/z) at a scan rate of 20 spectra/second. A predictable peak width of 3 s and a signal-to-noise (S/N) ratio of > 20 were used to detect peaks with 5 apexing masses.

### Miniaturised ^1^H‐NMR spectroscopy

^1^H‐NMR spectroscopy was carried out according to a miniaturised method previously described (Mason et al., 2018). Urine samples were analysed at 500 MHz in 2 mm NMR MATCH tubes, using the 5 mm triple-resonance inverse (TXI) probe head, which has been adjusted for ^1^H evaluation. During analysis a constant sample temperature was sustained at 300 K*.* ^1^H spectra were obtained as 128 transients in 32 K data points with a spectral width of 6000 Hz (12.0 ppm*).* A pulse sequence program NOESY-presat was used to supress the H_2_O resonance at 4.70 ppm, by saturating the H_2_O resonance by single-frequency irradiation through a 4 s relaxation delay, at a 90° excitation pulse of 10 μs. An acquisition time of 2.7 s and a receiver gain of 64 were set, correspondingly. The number of dummy scans (n = 4) and scans (n = 128) produced a run time of 15 min and 45 s per sample. Shimming was carried out for each sample automatically using the deuterium signal, then locked, probe tuned and matched, followed by pulse calibration. NMR analysis and processing was accomplished using Bruker Topspin (v 3.5), and additional processing was performed using Bruker AMIX (v 3.9.14).

### Inflammatory assay

To determine the inflammatory status in different dietary groups, a BD Cytometric bead array mouse inflammation kit (BD Biosciences, #552,364) and Accuri C6 flow cytometer (BD Biosciences) were used, according to the manufacturer instructions and the method discussed in the Supplementary material. The levels of selected inflammatory cytokines, namely, interleukin 6 (IL6), interleukin 10 (IL10), monocyte chemoattractant protein 1 (MCP1), tumour necrosis factor (TNF), interferon gamma (IFN-γ) and interleukin 12p70 (IL12p70) were determined.

### Data processing and statistical analysis

Data analysis for the growth rate, western blot analysis and inflammation assay was carried out using Excel 2021 (Microsoft 365). The average weight over the duration of the study was plotted on a bar graph for each group to visualise the growth rate. For the western blot results, the relative intensity of the target protein (p70S6K1) was normalised using the relative intensity of the control protein, β-actin, which was then plotted on a bar graph to observe the relative intensity differences between each group. The data obtained following the inflammation assay was pre-filtered manually using the FCAP Array software (v3.0), after which each cytokine was assigned to its relative peak intensity followed by manual clustering for each cytokine. The inflammation data of each cytokine’s relative concentrations were first plotted on a line graph to obtain a R^2^ value ≤ 1, then plotted on a bar graph to observe the differences of the cytokine concentrations between each group. Significant differences between groups for the growth rate, western blot and inflammation assay results were determined using a one-way ANOVA followed by a post-hoc Tukey’s test. The spectral data acquired from each metabolomics platform were extracted into matrices*.* Each data matrix was separately examined regarding correct peak selection and alignment, as well as batch effects and data integrity. The matrices were also pre-processed by which the aligned data was filtered to retain only features with an 80% presence within each group, along with removing data using a 50% cut-off coefficient of variation (CV) for quality-control samples in each group for GC-TOF-MS, LC–MS/MS and ^1^H-NMR. Urine data was normalised pre-acquisition to creatinine content (mg/g creatinine) and post-acquisition to tropic acid (GC-TOF–MS), isotope (LC–MS/MS) internal standards and TSP for data obtained via ^1^H-NMR analysis. LC metabolites were normalised using stable isotopes such as, valine_d8, isoleucine_d10, phenylalanine_d5, free carnitine_d3, octanoylcarnitine_d3, and octadecanoylcarnitine_d3 if there was a significant metabolite correlation to these isotopes. Batch corrections were not considered necessary, since QCs displayed no notable batch effects*.* Following this, log-transformation, mean centering and pareto scaling were applied for data pre-treatment and confidence intervals for PCA scores, confirmed by cluster analysis, were used for outlier detection. The data underwent both multivariate and univariate analyses*.* Initially, all groups (normal diet vs normal diet with alcohol vs KD vs KD with alcohol) were compared visually using PCA scores plots to observe within group variances and whether the data could partition animals into the different groups. Following this*,* univariate analysis using one-way ANOVA, along with a post-hoc Tukey’s test, were applied to shortlist differentiating compounds between groups. A two-way ANOVA was used to evaluate diet and alcohol’s interaction effects for the various compounds for each group, where a significant interaction was evaluated by a subsequent post-hoc Tukey’s test. Metabolites (features) were deemed significant if they had a false discovery rate-corrected p-value (q-value) of ≤ 0.05, using a post-hoc Tukey’s test, and a Cohen’s d-value of ≥ 0.8 (Ellis & Steyn, 2003). Both the PCA and one-way ANOVA were performed in MetaboAnalyst 5.0 (Xia et al., 2015), whereas the two-way ANOVA, along with a subsequent post-hoc Tukey’s test, was carried out using IBM SPSS Statistics 27 and Graphpad Prism 9. Lastly, if spectral features had high identification confidence levels, they were assigned metabolite identities (Schymanski et al., 2014). Commercial and in-house spectral libraries, in conjunction with public databases, were used to identify dataset features through spectral and retention time matching. The significant features’ identities were confirmed using each platform’s relative standards as well as isotopes (Schymanski et al., 2014).These features were also subjected to pathway analysis using a manual approach, to identify pathways of significance.

## Results and discussion

### Growth curves

**Fig. 1:**
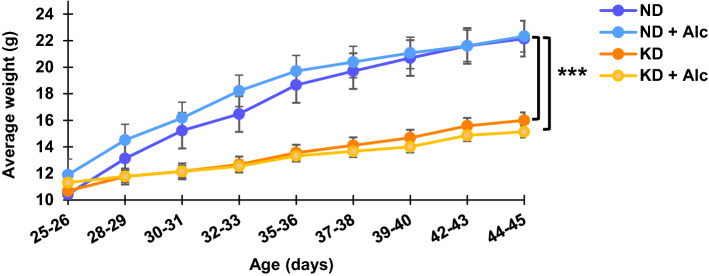
Consumption of a KD in mice leads to lower body weight gain. Growth curves for dietary groups on a normal diet without (ND) and with alcohol intake (ND + Alc.) and on a ketogenic diet without (KD) and with alcohol intake (KD + Alc.). Average weight ± standard deviation (n = 10) per group is given as a function of animal age. Mice were weighed three times a week. Asterisks (***p < = 0.001) indicate significant differences between ND vs KD and ND vs KD + Alc. groups as determined by post-hoc Tukey’s test

Growth curves (Fig. 1) were constructed to evaluate the effect of a KD and chronic alcohol (Alc.) consumption on animal body weight over a period of three weeks. Mice on a KD had a significantly reduced growth rate relative to those on a control diet (ND), with the final average body weight being 22 ± 2 g for the ND and ND + Alc. groups and 15 ± 2 g for the KD and KD + Alc. groups. Therefore, it is evident that diet alone (i.e., a KD) had a significant (*p* < 0.001) effect on murine body mass, while chronic alcohol consumption did not. The available literature on the effect of alcohol on body weight is conflicting, as studies show either an increase, decrease or no difference at all of alcohol consumption on body weight (Traversy G, Chaput, 2015; Huang et al., 2012; Perides et al., 2005). On the contrary, several studies have confirmed that a KD reduces body weight gain, possibly due to mTOR inhibition (Bueno et al., 2013; Roberts et al., 2017; Santos et al., 2012).

### mTOR activity

**Fig. 2:**
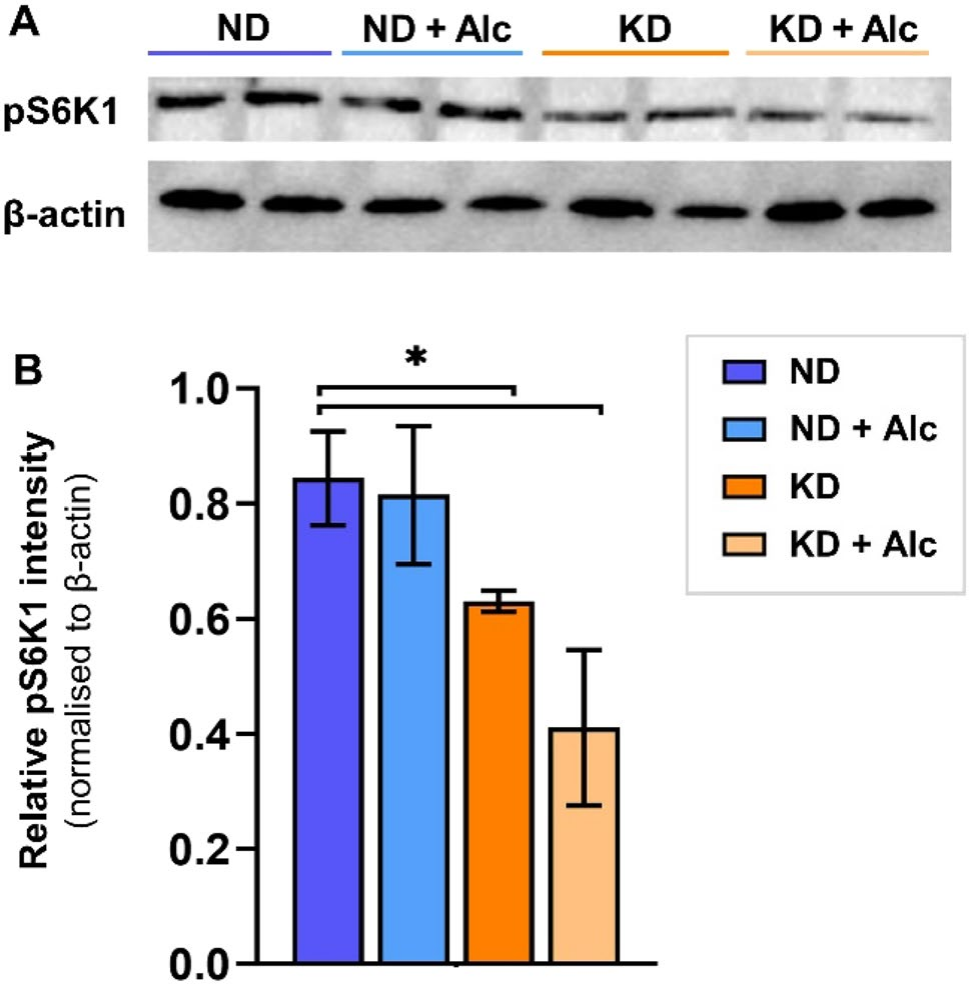
A KD inhibits S6K1 phosphorylation in mice. **A** Representative Western blot image and **B** quantitative summary of pS6K1 and β-actin following separation by SDS-page. Average β-actin (~ 42 kDa) was used as control protein of pS6K1 (~ 75 kDa). Data represents the average relative pS6K1 intensity ± standard deviation (n = 4) per group. ND indicates normal diet; ND + Alc., normal diet and alcohol; KD, ketogenic diet; KD + Alc., ketogenic diet and alcohol. Asterisks (*p < 0.05) indicate significant differences between ND vs KD and ND vs KD + Alc. Groups, as determined by post-hoc Tukey’s test

The mTOR substrate, S6K1 (Thr389), is frequently used as a viable indicator of mTORC1 activity (Ikenoue et al., 2009). Therefore, we examined liver S6K1 phosphorylation status in the various groups using western blot analysis. Liver pS6K1 levels (Fig. 2) were significantly diminished (p < 0.05) in the KD and KD + Alc. groups compared to mice on a normal diet (ND). Hence, we confirm that a KD significantly inhibits mTOR pathway activity in mice, an observation supported by previous research (McDaniel et al., 2011). Chronic alcohol consumption alone (ND + Alc. group), however, did not significantly alter murine S6K1 phosphorylation status, with pS6K1 levels only trending lower. Additionally, no significant difference was observed between the KD + Alc. group and the KD group. In addition, no significant interaction was observed between diet and alcohol for mTOR activity. As such, it does not seem that chronic alcohol consumption alone or alcohol consumed along with a KD has a significant impact on mTOR activity. A KD significantly lowered growth rates and levels of liver pS6K1, a downstream substrate of mTORC1 phosphorylation, in mice. These results support previous studies showing that a KD inhibits mTOR phosphorylation (McDaniel et al., 2011) and has a considerable impact on the management of weight and obesity (Bueno et al., 2013; Roberts et al., 2017; Santos et al., 2012). In contrast, chronic alcohol consumption did not markedly affect growth rates or S6K1 phosphorylation when consuming a normal or KD, despite previous reports of its potential inhibitory effect on the mTOR pathway (Foster, 2010). These results are in agreement with previous studies that did not notice any significant changes in growth from alcohol consumption (Perides et al., 2005). However, since the literature regarding chronic alcohol consumption’s effect on weight is conflicting (Traversy G, Chaput, 2015; Huang et al., 2012; Perides et al., 2005), further research is warranted. It is well known that mTORC1 activation is essential for growth (Wullschleger et al., 2006), with reduced phosphorylation of this pathway correlated to impaired cell growth (Laplante et al., 2012). Thus, the data confirms that a KD reduces growth rate, possibly through mTOR inhibition, while chronic alcohol consumption does not markedly impact mTOR activity or body weight in mice. Moreover, the results obtained from the study indicate that the combination of a KD and chronic alcohol consumption does not significantly inhibit mTOR to a greater extent than a KD alone.

## Metabolic alterations

**Fig. 3:**
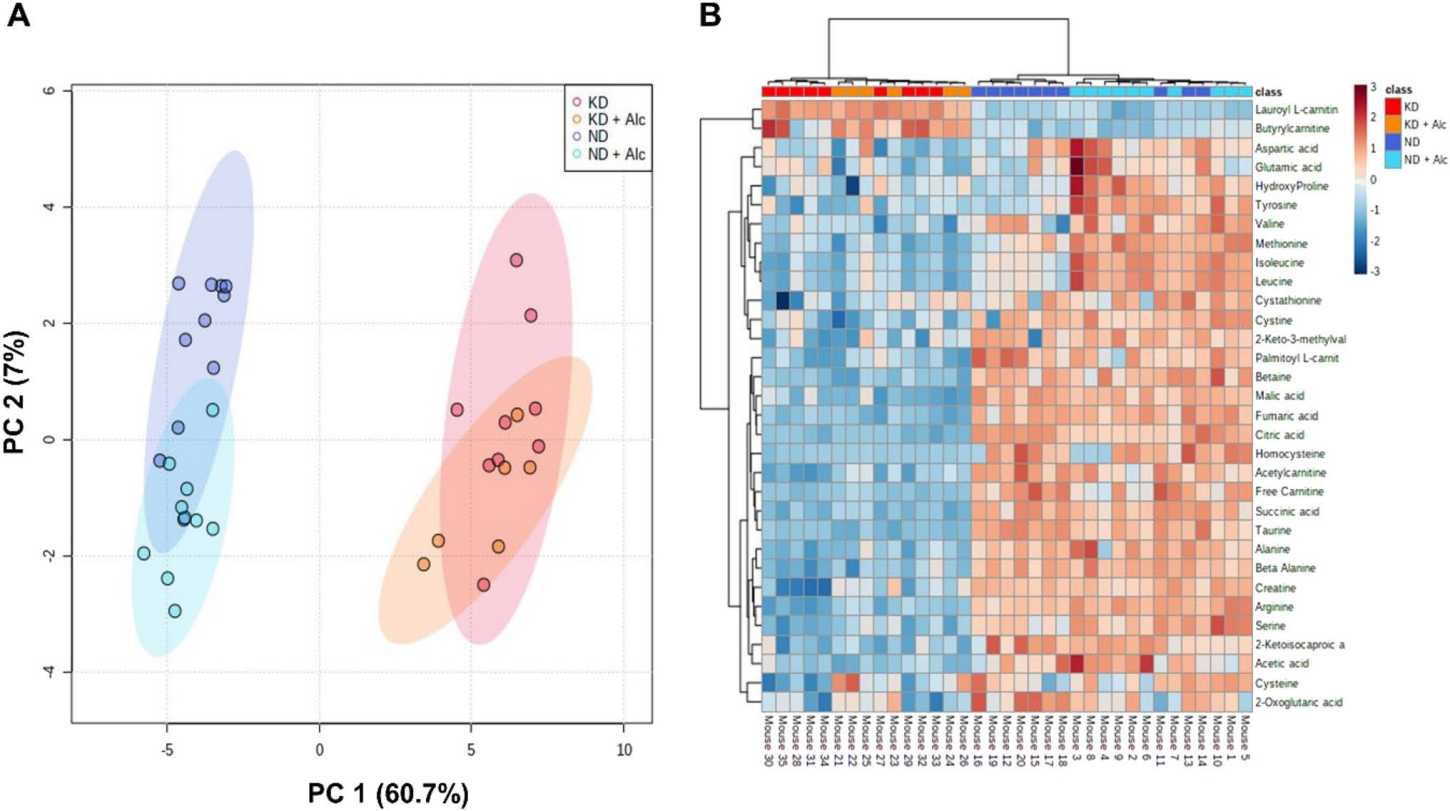
A KD and chronic alcohol consumption leads to metabolic alterations in mice. **A** PCA scores plot illustrates the natural separation of all significantly altered metabolites when the different groups (n = 6 to 10 per group) were compared. **B** Heatmap of main metabolites altered in different groups (n = 6 to 10 per group) following dietary intervention. Each metabolite was normalised to its respective internal standard (based on the relative metabolomics platform) and subsequently log transformed, as well as auto-scaled using MetaboAnalyst 5.0

**Fig. 4:**
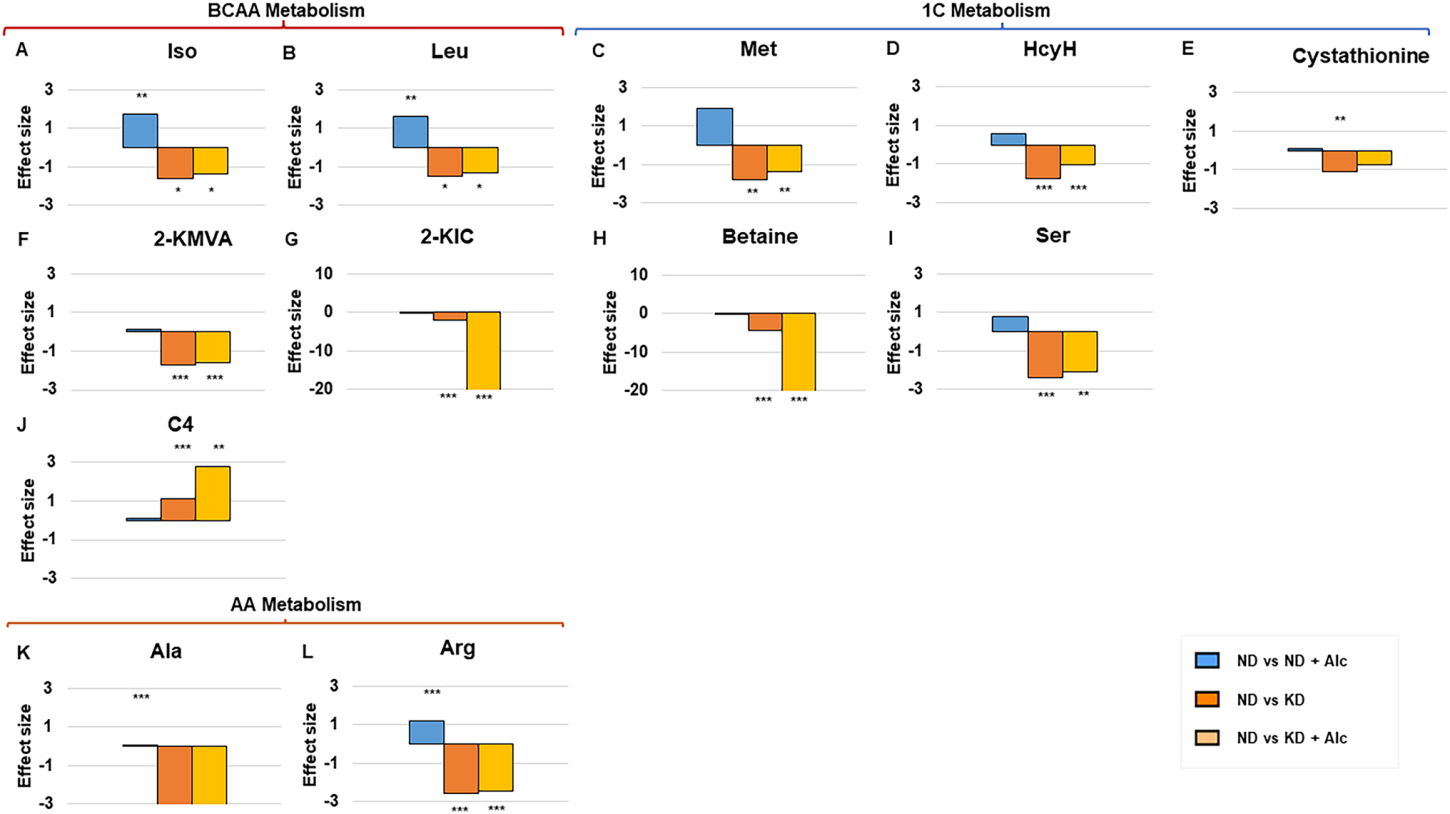
A KD lowers urinary amino acid levels irrespective of alcohol consumption. Bar graphs depict Cohen’s *d*-values (effect size) for each metabolite when relative abundance between the different mouse groups (n = 6 to 10 per group) were compared to the control group (ND). *Leu indicates leucine; Ile, isoleucine; 2-KIC, 2-ketoisocaproic acid; 2-KMVA, 2-keto-3-methylvaleric acid; C4, butyryl-carnitine; Arg, arginine; Ala, alanine; Met, Methionine; HcyH, homocysteine; Ser, serine; ND, normal diet; ND* + *Alc., normal diet and alcohol; KD, ketogenic diet; KD* + Alc., ketogenic diet and alcohol. *p < 0.05, **p < 0.01, ***p < *0.001* vs. the control group, obtained via a post-hoc Tukey’s test

**Fig. 5:**
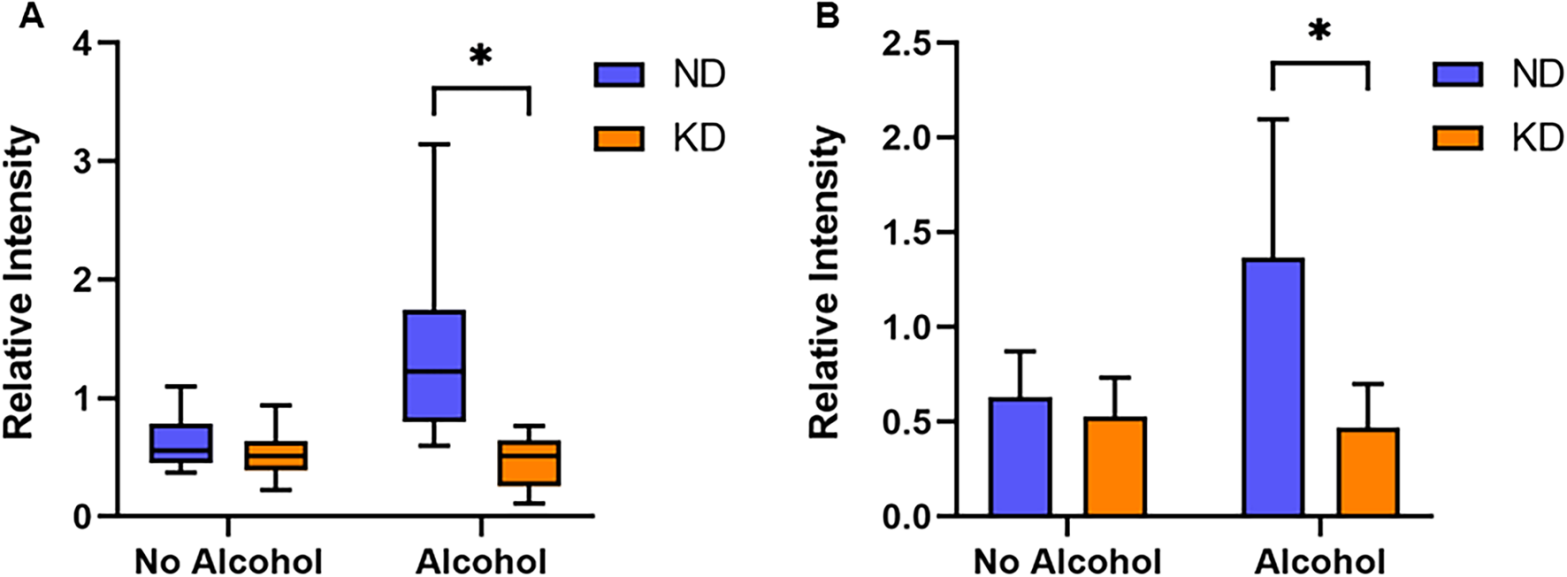
A KD prevents increased urinary 4-hydroxyproline excretion observed during chronic alcohol intake. **A** Boxplots depict the relative 4-hydroxyproline intensity for dietary groups (n = 6–10 per group) on a normal diet without (ND) and with alcohol intake (ND + Alc.) and on a ketogenic diet without (KD) and with alcohol intake (KD + Alc.). For each boxplot the length of the box represents the 25th to 75th percentile inter-quartile range, the interior horizontal line represents the median, and the vertical lines stemming from the box extend to minimum and maximum values. **B** Bar graphs show the relative intensities for 4-hydroxyproline when the interaction of diet and alcohol was compared. Asterisks (*p < 0.05) indicate significant differences between the ND vs ND + Alc. groups as determined by post-hoc Tukey’’s test

Next, we investigated the systemic effect of a KD and chronic alcohol consumption on murine metabolism via GC-TOF-MS, LC–MS/MS and ^1^H-NMR spectroscopy. ANOVA with post-hoc Tukey’s test identified numerous metabolites significantly differing among the four dietary intervention groups (n = 6–10 per group, Table S3). Multivariate principal component analysis (PCA; Fig. 3A) and a heatmap (Fig. 3B) of these discriminatory metabolites revealed a distinctive metabolic separation between animal groups on a KD and control diet. However, chronic alcohol consumption could only partially separate animal groups on the same diet, indicating that diet (i.e., a KD) had a greater impact on metabolism than alcohol consumption. When comparing the urinary metabolomes of the different dietary intervention groups to controls (ND), significant alterations were observed in amino acid and TCA cycle metabolism. Subsequent pathway analysis revealed that the most significantly altered urinary metabolites were involved in amino acid metabolism (Fig. 4), with branched-chain amino acid and one-carbon metabolism markedly affected, and the tricarboxylic acid (TCA) cycle (Fig. 5). Figures 4–5 illustrate the practical (effect size d-value) and statistical (p-value) significance of these metabolic alterations in the KD, KD + Alc. and ND + Alc. groups when respectively compared to controls (ND). Other metabolites that also displayed significant interaction effects were creatine, alloxanoic acid and isobutyric acid. Additional information on metabolites with an interactive effect can be found in the Supplementary material (Table S4 and Fig. S3). All significantly altered features are described in the Supplementary material.

### Amino acid metabolism

Several essential (leucine, isoleucine, arginine, and methionine), non-essential (alanine, aspartate and serine) and non-proteinogenic (homocysteine and cystathionine) amino acids were markedly (p < 0.001 and d > 0.8) reduced in the urine of animals consuming a KD with and without alcohol when compared to controls (ND). Additionally, leucine and isoleucine catabolites, 2-ketoisocaproic acid and 2-keto-3-methylvaleric acid along with choline catabolite, betaine, were also significantly lower in these mice (KD and KD + Alc.) compared to controls. Contrastingly, animals chronically consuming alcohol while on a normal diet (ND vs ND + Alc.) excreted markedly higher (p < 0.001 and d > 0.8) levels of essential amino acids, leucine, isoleucine, arginine, and methionine along with proline’s post translational product, 4-hydroxyproline (Fig. 5). The latter serves as a biomarker for bone resorption and is used to detect osteoporosis (Barbul, 2008; Kuo and Chen, 2017). Interestingly, 4-hydroxyproline levels were unaltered in mice consuming alcohol while on a KD. Overall, a KD more dramatically altered amino acid metabolism than alcohol consumption. When the amino acid metabolites were evaluated for an interactive effect between diet and alcohol only a few metabolites were observed to have interaction, namely, taurine (conditionally essential), cysteine (non-essential) and 4-hydroxyproline. Mice fed a KD diet (KD and KD + Alc.) excreted significantly lower levels of several amino acids, including the essential amino acids, leucine, isoleucine, arginine, and methionine along with several other amino acids that form part of one-carbon metabolism (homocysteine, serine, cystathionine, and betaine). This reduction in amino acid levels could, in part, reflect decreased protein intake, since a KD is inherently low in protein (8.2% of calories from protein in KD vs 20% in ND). However, it was recently shown that a KD with at least 5% of calories derived from protein does not induce a protein restriction response (Hu et al., 2018). We therefore postulate that the marked reduction observed in mouse urinary one-carbon metabolites while on a KD, reflect a perturbation in this pathway. In support, others have shown that a KD results in the downregulation of a subset of genes involved in one-carbon metabolism in mouse liver (Pissios et al., 2013) and an inhibition of one-carbon metabolism in HepG2 cells and mice (Hsu et al., 2022). This inhibitory effect on one-carbon metabolism has been ascribed to the reduced mTOR signalling associated with a KD (Hsu et al., 2022). Chronic alcohol intake during a ND significantly increased the excretion of the same essential amino acids lowered by a KD, likely reflecting altered protein turnover (Bernal et al., 1993; Lang et al., 2010). However, alcohol consumption did not markedly alter the effect of a KD on amino acid metabolism. In addition, one of the most significant metabolic alterations resulting uniquely from chronic alcohol consumption, was an increase in urinary 4-hydroxyproline levels. 4-Hydroxyproline is a major part of collagen, with urinary levels thereof serving as a bone-resorption biomarker of collagen turnover (Barbul, 2008; Kuo and Chen, 2017). Hence, the observed elevation in 4-hydroxyproline may reflect increased collagen degradation, a phenomenon previously linked to chronic alcohol consumption (Preedy et a., 1991). Importantly, animals chronically consuming alcohol while on a KD, did not display any alterations in 4-hydroxyproline levels. Thus, a KD seems to play a protective and preventative role in alcohol-induced collagen degradation. However, further research is warranted, as we only assessed 4-hydroxyproline, while several other biomarkers of collagen turnover exist (Preedy et a., 1991).

### TCA cycle metabolism

**Fig. 6:**
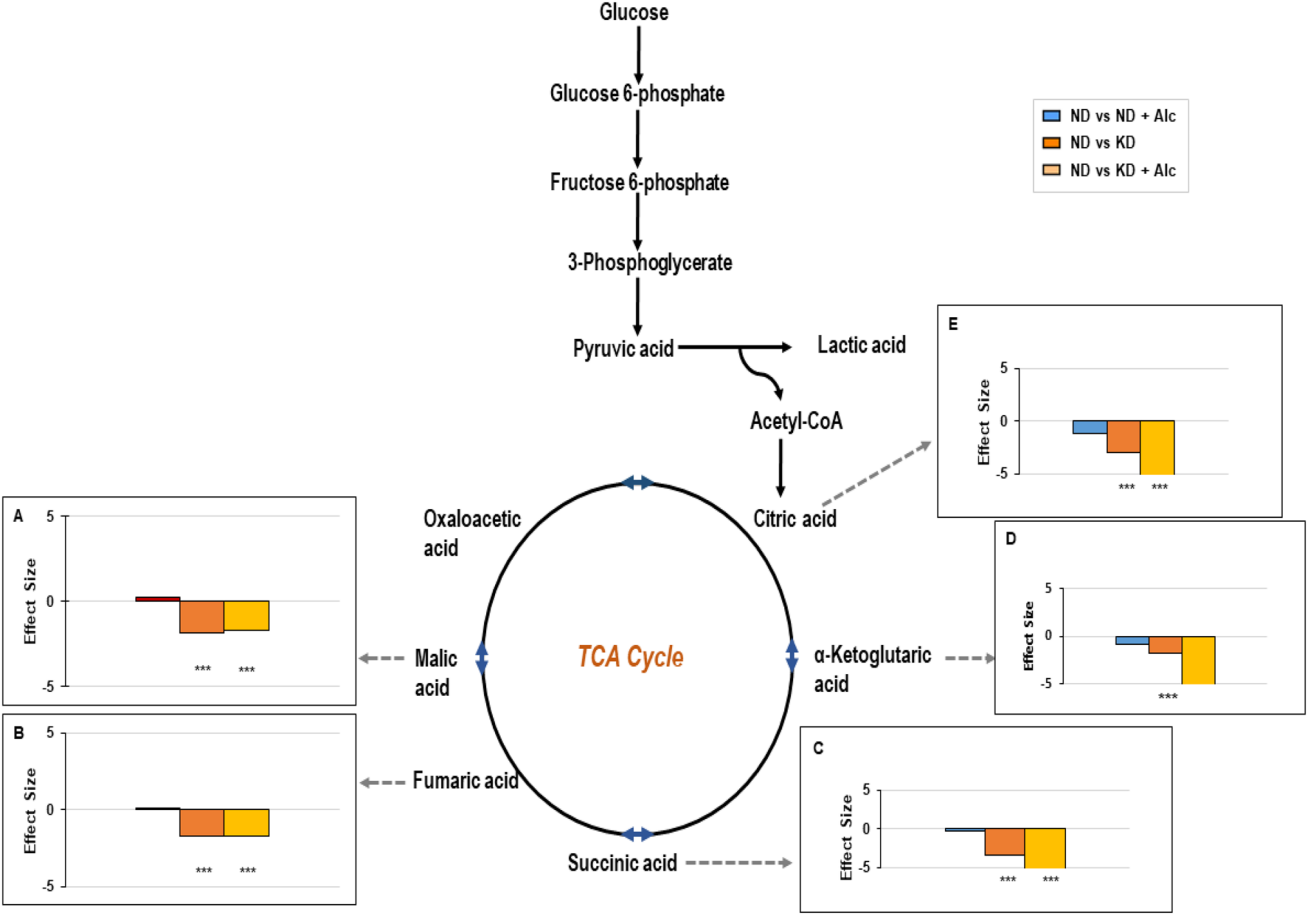
Alterations of TCA cycle intermediates are more prominent with dietary interventions than chronic alcohol consumption when the different mouse groups (n = 6–10 per group) were compared. Bar graphs reveal Cohen’s *d*-values (effect size; *d* > 0.8) for the significant metabolites when the relative abundance between the different mice groups were compared to the control group (ND). ND indicates normal diet; ND + Alc., normal diet and alcohol; KD, ketogenic diet; KD + Alc., ketogenic diet and alcohol. ***p < 0.001 vs. the control group, obtained via a post-hoc Tukey’s test

TCA cycle intermediates, malic acid (p < 0.001 and d > 0.8; Fig. 6A), fumaric acid (p < 0.001 and d > 0.8; Fig. 6B), succinic acid (p < 0.01 and d > 0.8; Fig. 6C), α-ketoglutaric acid (p < 0.001 and d > 0.8; Fig. 6D) and citric acid (p < 0.001 and d > 0.8; Fig. 6E) were significantly decreased in the KD and KD + Alc. groups compared to controls. It was also noted that for the KD + Alc. group, α-ketoglutaric acid and citric acid showed the greatest practical significance when the effect size was assessed compared to the control. Other studies using a KD have made similar observations and link this decrease in TCA cycle intermediates to the inhibitory effect of a KD on mTOR activity (Longo et al., 2019; Ramanathan & Schreiber, 2009). This corresponds to the mTOR inhibition we observed in mice on a KD via Western blotting. Additionally, chronic alcohol consumption did, however, seem to exacerbate citric acid, α-ketoglutaric acid and succinic acid depletion in mice on a KD. In accord, previous studies have also shown that chronic alcohol consumption can lead to decreased levels of TCA cycle intermediates (Chen et al., 2012). Hence, it is evident that the metabolism of the TCA cycle is significantly altered by a KD and altered to an even greater extent when alcohol is consumed in conjunction, which ultimately results in alterations of energy metabolism and the redox state (Auta et al., 2017).

### Redox state evaluation via metabolic indicators of NADH/NAD^+^ ratio

**Fig. 7:**
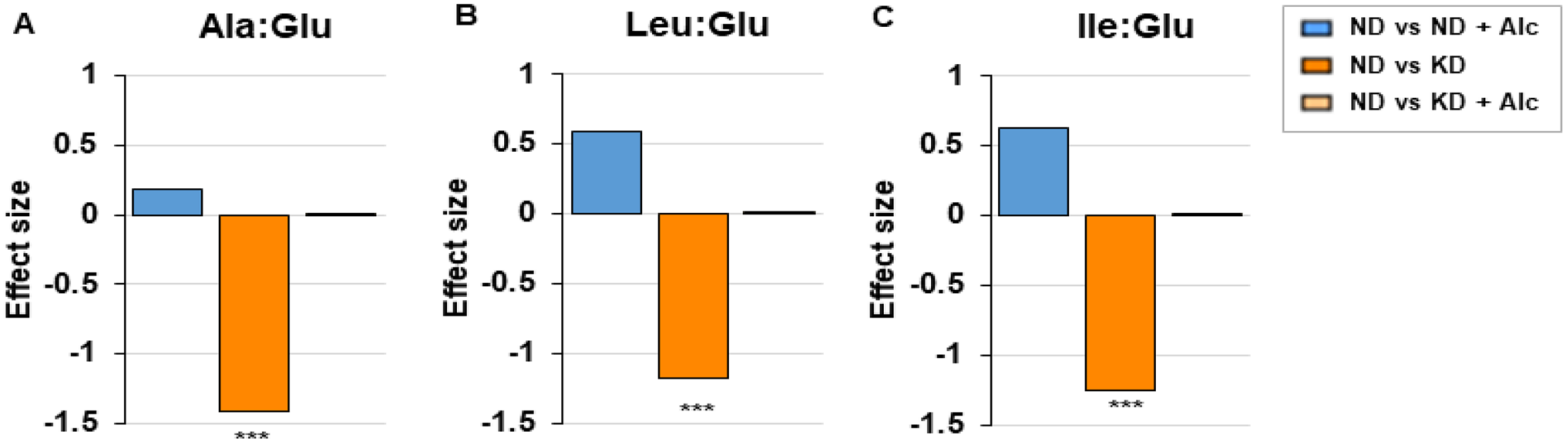
A KD results in a more oxidative redox state in mice. Bar graphs (A–C) depict Cohen’s d-values (effect size) of alterations in metabolic indicators of NADH/NAD^+^ ratio, when the different dietary groups were compared to controls. Positive and negative effect size values respectively indicate increased and decreased levels in the experimental group vs the control group. An elevated NADH/NAD^+^ ratio drives the formation of amino acids from glutamic acid. Ala indicates alanine; Glu, glutamic acid; Ile, isoleucine; Leu, leucine; ND, normal diet; ND + Alc., normal diet and alcohol; KD, ketogenic diet; KD + Alc., ketogenic diet and alcohol. Asterisks (***p < = 0.001) indicate significant differences between the ND vs KD group as determined by post-hoc Tukey’s test

**Fig. 8:**
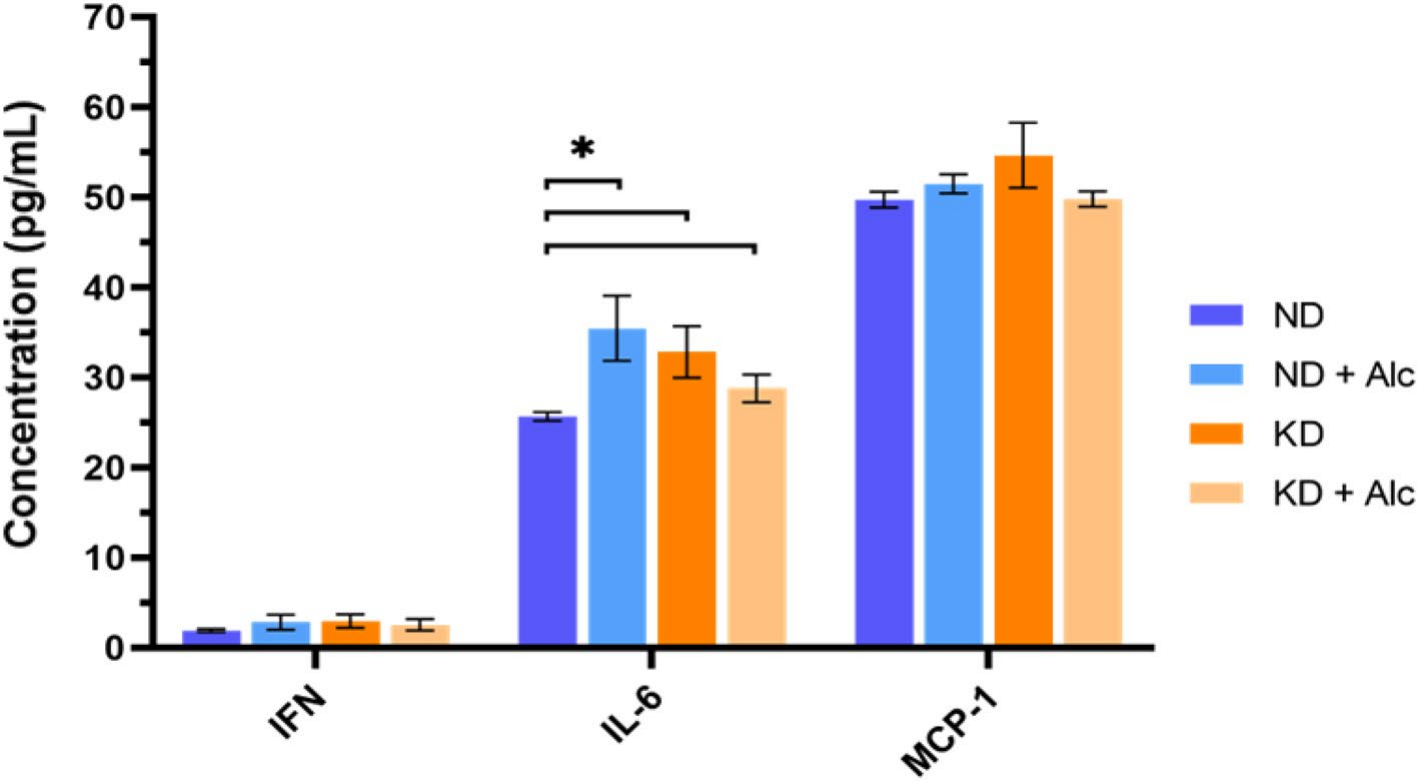
The concentration levels (pg/mL) of pro-inflammatory biomarkers as measured in the serum of mice from all four dietary groups (n = 6 per group). The error bars represent the standard error. ND indicates normal diet; ND + Alc., normal diet and alcohol; KD, ketogenic diet; KD + Alc., ketogenic diet and alcohol. *p < 0.05 vs. the control group, obtained via a post-hoc Tukey’s test,

To evaluate the effect that either a KD or chronic alcohol consumption has on the redox status in mice, metabolic indicators of NADH/NAD^+^ ratio were assessed in urine (Fig. 7), as it is difficult to detect NAD^+^ and NADH by conventional metabolomics (Smith et a., 2019). Metabolic indicators that are usually used include alanine, leucine, and isoleucine in relation to glutamate (Smith et a., 2019). Seeing that an elevated NADH/NAD^+^ ratio drives the formation of amino acids from glutamic acid (i.e., an increase in amino acid production from glutamic acid is correlated to increased NADH levels) (Smith et al., 2019), the ratio of various amino acids relative to glutamic acid were assessed. The ratios of alanine (p < 0.001; Fig. 8A), isoleucine (p < 0.001; Fig. 8B) and leucine (p < 0.01; Fig. 8C) relative to glutamic acid were significantly decreased in the group consuming a KD solely. The consumption of alcohol on a ND lead to trended higher levels of amino acid ratios, indicating a more reduced redox state in mice. However, no statistically significant differences were observed for the groups consuming alcohol on either the control diet (ND + Alc.) or a KD (KD + Alc.). Thus, it is evident from the mentioned findings that mice consuming a KD had a more oxidative redox state (i.e., a decreased level of NADH relative to NAD^+^) compared to the control diet (ND), while the other groups had no significant alteration in redox state. The mentioned amino acid metabolite ratios indicated a significantly decreased NADH/NAD^+^ ratio in mice on a KD compared to controls (ND). Thus, confirming that a KD increases the availability of NAD^+^ through altering the NADH/NAD^+^ ratio. This alteration in redox balance could be related to the observed perturbations in amino acid and energy metabolism in mice on a KD diet. No significant alterations in NADH/NAD^+^ ratio was seen following alcohol consumption in the normal diet group (ND + Alc.), indicating that diet alone (i.e., KD) had a greater impact on the redox state than alcohol consumption. However, chronic alcohol consumption along with a KD, prevents any favorable changes towards a decreased NADH/NAD^+^ ratio, which would be observed when a KD is consumed alone. Previous research has shown that the metabolism of alcohol results in redox state changes due to an increase in the NADH/ NAD^+^ ratio (Cederbaum, 2012). On the contrary, other studies have shown that a KD can decrease the NADH/NAD^+^ ratio, thereby altering the redox state (Elamin et al., 2017; Xin et al., 2018). As such, our findings provide insight with regards to changes in the redox state when alcohol is consumed together with a KD.

### Inflammatory status of mouse serum

Several serum inflammatory biomarkers were measured in the different dietary groups. All experimental groups displayed significantly increased (p < 0.05; Fig. 8) levels of IL-6 relative to the control group. The ND + Alc. group had the highest increase in IL-6, followed by the KD group and then the KD + Alc. group. This finding suggests that both alcohol and a KD has a pro-inflammatory effect. However, no significant alterations in IFN or MCP-1 levels were observed among the dietary groups, while marked intragroup variation limited our use of the other measured inflammatory cytokines (Fig. S4). Although several studies have described the anti-inflammatory properties of a KD (Ciaffi et al., 2021; Dupuis et al., 2015), others have also observed signs of increased inflammation associated with a KD in mice (Garbow et al., 2011) and human subjects (Rosenbaum et al., 2019). Thus, no conclusive assumption can be made with regards to a KD and chronic alcohol consumption’s impact on inflammation and further research is required. Moreover, seeing that existing literature also shows conflicting evidence with regards to the effect a KD and alcohol consumption has on inflammation (Ciaffi et al., 2021; Dupuis et al., 2015; Garbow et al., 2011; Rosenbaum et al., 2019). Considering our inconclusive observations, along with conflicting literature reports, further research on the inflammatory effects of a KD and alcohol consumption is warranted.

## Conclusion

The therapeutic potential of a KD and its mechanisms of action for conditions such as, epilepsy, obesity, diabetes, inflammation, and mitochondrial disease has gained immense attention in recent years (Vinnig, 1999; Qu et., 2021; Bolla et al., 2019; Freeman et al., 2010; Dupuis et al., 2015; Jin et al., 2014). The regulatory effect that a KD has on the mTOR pathway is of great importance (Genzer et al., 2016) and has been evaluated in various disease models (Genzer et al., 2016; Johnson et al., 2013; McDaniel et al., 2011; Roberts et al., 2017; Singh et al., 2018; Tian et al., 2019; Xie et al., 2016; Zeng et al., 2008). Since alcohol consumption reportedly alters the cellular redox state and impedes mTOR phosphorylation (Feldstein & Bailey, 2011; Lang et al., 2003; Zakhari, 2006), we aimed to establish the effect that chronic alcohol consumption has on the mTOR pathway and systemic metabolism during a KD-based intervention*.* This was achieved by investigating the isolated and combined effects of chronic alcohol consumption and a KD on growth rate, S6K1 phosphorylation, urinary metabolism, metabolic indicators of redox state, and serum inflammatory markers in a healthy mouse model. This study, however, did have its limitations. Firstly, alcohol and food intake were not monitored accordingly when mice were given ad libitum access to alcohol drinking water and food. As such, it is possible that the mice on a KD and those on a KD with chronic alcohol consumption consumed less food than that of the control diet, especially since a KD has previously been linked to increased satiety and decreased food intake (Paoli et al., 2015). In addition, this could also lead to the mice consuming less vitamins and trace minerals, which in return could contribute to difference in growth (Churuangsuk et al., 2019; Kenig et al., 2019). Additionally, the KD used in this study was not an isocaloric KD, as the total calorie content was not identical for the ND and KD. Moreover, the KD used in this study was also more protein-restricted when compared to the ND. Lastly, consideration needs to be taken when a similar dietary strategy is applied to humans, since the mice were three weeks old when they were put on the dietary intervention. Children might respond differently than adults when put on a ketogenic dietary intervention (Kim et al., 2019).

In conclusion, this study shows that a KD attenuates mTOR signalling and weight gain, as well as decreases the excretion of amino acid and TCA cycle metabolites in mice. Also, the KD seems to drive energy metabolism towards a more oxidative redox state by increasing the NAD^+^/NADH ratio through metabolic regulation. In addition, the study’s results regarding a KD’s effect on inflammation were inconclusive due to limited sample volume and the fact that only one pro-inflammatory marker, IL-6, was significantly increased. As such, more research regarding a KD’s effect on inflammation is warranted. Furthermore, we provide evidence that a KD could decrease or prevent bone loss and collagen degradation, by decreasing structural protein degradation even during chronic alcohol consumption. Although findings of previous studies have shown that a KD alone does not lead to potential bone loss and degradation (Bisschop et al., 2003), our study is the first to report that a KD could potentially reduce bone loss and collagen degradation during chronic alcohol consumption. As such, it is evident that a KD indeed has some therapeutic potential, especially for conditions that lead to an unfavourable redox state, increase in structural protein degradation, as well as disorders in which attenuation of mTOR leads to improved disease outcome. However, the potential benefits of a KD could be overturned when alcohol is consumed chronically in conjunction with a KD, as alcohol consumption seems to prevent the shift towards a more oxidative redox state and lead to a potential decrease in NAD^+^ levels. Thus, any intervention using a KD for the management of conditions like obesity, diabetes, epilepsy, and other metabolic disorders, should take note of the factors that could potentially hinder the therapeutic effects of a KD, such as chronic alcohol consumption, especially where the redox state is of concern. Further research is warranted making use of multiple protein markers to monitor mTOR activity, as well as applying multi-platform metabolomics on other tissues such blood, brain, and muscle to evaluate tissue specific differences in systemic metabolism, to further assess the therapeutic potential of a KD. Lastly, any study looking at the effect of a KD should also consider the total protein and total caloric content of the diet, as well as the effects it has on the dietary intervention.

## Supplementary Information

Below is the link to the electronic supplementary material.

Supplementary file1 (DOCX 886 KB)

## Data Availability

The datasets are available from the corresponding author upon reasonable request.

## Abbreviations

KD: Ketogenic diet
mTOR: Mammalian target of rapamycin
LC–MS/MS: Liquid chromatography mass spectrometry/mass spectrometry
GC-TOF–MS: Gas chromatography time of flight mass spectrometry
1H-NMR: Proton nuclear magnetic resonance
