# Supplementary material for: A ketogenic diet alters mTOR activity, systemic metabolism and potentially prevents collagen degradation associated with chronic alcohol consumption in mice
Source: Metabolomics. 2023 Apr 19;19(5):43. doi: 10.1007/s11306-023-02006-w (PMC10115735; doi:10.1007/s11306-023-02006-w)
Supplement: Supplementary file 1 — Supplementary file1 (DOCX 886 KB) [file 11306_2023_2006_MOESM1_ESM.docx]

**A ketogenic diet alters mTOR activity, systemic metabolism and potentially prevents collagen degradation associated with chronic alcohol consumption in mice**

Luciano Willemse, Karin Terburgh, Roan Louw^1^

*Human Metabolomics, Faculty of Natural and Agricultural Sciences, North-West University (Potchefstroom Campus), South Africa*

**Supplementary Information**

1. **Materials and methods**
   1. **Dietary Composition**

| **A**   \| **Control diet nutritional value** \| **“As is” (g/kg)** \| **DM (g/kg)** \| \| --- \| --- \| --- \| \| **Protein (min)** \| 220 \| 240 \| \| **Moisture (max)** \| 100 \| **-** \| \| **Oils and fats (min)** \| 50 \| 55 \| \| **Linoleic acid (min)** \| 12 \| 14 \| \| **Fiber (max)** \| 40 \| 45 \| \| **Ash (max)** \| 70 \| 75 \| \| **Ca:P ratio** \| 1.1-2:1 \| 1.1-2:1 \| \| **Calcium (min)** \| 12 \| 14 \| \| **Phosphorous (min)** \| 7.5 \| 8 \| \| **Vitamin A (min)** \| 16 000 (IU/kg) \| 16 000 (IU/kg) \| \| **Vitamin D (min)** \| 2000 (IU/kg) \| 2000 (IU/kg) \| \| **Vitamin E (min)** \| 100 (g/kg) \| 100 (g/kg) \| \|  \|  \|  \|   **Ingredients**: Maize, wheat bran, soybean, soybean protein concentrate, fish meal, maize  protein concentrate molasses, sucrose, calcium carbonate, sodium chloride, calcium phosphate  **B**   \| **Ketogenic diet nutritional value** \| **“As is” (g/kg)** \| **DM (g/kg)** \| \| --- \| --- \| --- \| \| **Protein (min)** \| 121.5 \| 135 \| \| **Oils and fats (min)** \| 750 \| 790 \| \| **Linoleic acid (min)** \| 320 \| 360 \| \| **Fiber (max)** \| 55 \| 60 \| \| **Ca:P ratio** \| 1.1-2:1 \| 1.1-2:1 \| \| **Calcium (min)** \| 12 \| 14 \| \| **Phosphorous (min)** \| 7.5 \| 8 \| \| **Vitamin A (min)** \| 16 000 (IU/kg) \| 16 000 (IU/kg) \| \| **Vitamin D (min)** \| 2000 (IU/kg) \| 2000 (IU/kg) \| \| **Vitamin E (min)** \| 100 (g/kg) \| 100 (g/kg) \| \|  \|  \|  \|   **Ingredients:** Palm oil, butter, canola oil, casein, cellulose, dextrose, vitamin & mineral mix  **C**   \|  \| **Control diet (%cal per 100g)** \| **Ketogenic diet (%cal per 100g)** \| \| --- \| --- \| --- \| \| **Protein** \| 20 \| 8.2 \| \| **Fat** \| 5 \| 91.7 \| \| **Carbohydrate** \| 75 \| <0.1 \| |
| --- | --- | --- | --- | --- | --- | --- | --- | --- | --- | --- | --- | --- | --- | --- | --- | --- | --- | --- | --- | --- | --- | --- | --- | --- | --- | --- | --- | --- | --- | --- | --- | --- | --- | --- | --- | --- | --- | --- | --- | --- | --- | --- | --- | --- | --- | --- | --- | --- | --- | --- | --- | --- | --- | --- | --- | --- | --- | --- | --- | --- | --- | --- | --- | --- | --- | --- | --- | --- | --- | --- | --- | --- | --- | --- | --- | --- | --- | --- | --- | --- | --- | --- | --- | --- | --- | --- | --- | --- | --- | --- |

**Table S1.** Nutritional profile and ingredient composition of the control and ketogenic diets. Cal, calories; DM, dry matter; g/kg, gram per kilogram.

- 1. **SDS-PAGE analysis and western blotting**

Liver tissue was homogenized in HEPES lysis (~1 mL per 100mg tissue) buffer via a 10 mL Glass/Teflon® Tight Fitting Potter-Elvehjem Homogeniser (Glas-Col, USA) using 15 strokes. Following homogenisation, the liver homogenates were centrifuged at 1000 x g for 10 min at 4 °C with a Heraeus™ Multifuge™ X3 centrifuge (Thermo Fisher Scientific, USA). Subsequently, the supernatants were collected, and the protein concentrations were determined with a BCA protein assay. Samples were prepped for SDS-PAGE by mixing the sample with 4X laemmli sample buffer and milli-Q water to a final volume of 25 µL, which was then boiled at 100ºC for 5 min. For each dietary group, aliquots (20 μg) of the protein were separated via SDS-PAGE at 300V for 22 min using a 10% acrylamide gel, then transferred to nitrocellulose membranes (cat #230-4, Bio-Rad, USA), and blocked using 1% casein in TBST buffer (cat #161-0782, Bio-Rad, USA) for 1 h*.* After that, the membranes were incubated with phospho-p70 S6 kinase primary antibodies (cat #9234S, Cell Signalling Technologies, USA) and the control protein, β-actin (cat # ab6276, Abcam, UK), at 4 °C overnight. Afterwards, the membranes were incubated with secondary antibodies (cat # ab97051, Abcam, UK) at room temperature for 1 h. Then, chemiluminescent detection was achieved using a Western ECL substrate (cat #170-5060, Bio-Rad, USA) and the ChemiDoc^TM^ MP imaging system (Bio-Rad, USA)*.* Finally, the results were analysed via Image Lab^TM^ v. 6.1 software (Bio-Rad, USA) in order attain the density ratio of the target proteins relative to β-actin.

Different approaches for normalizing phospho-p70S6k exists, but we derived our normalization approach using β-actin from previous studies making use of the same antibody (Hsin et al., 2021; Wu et al., 2021) although normalization against the total p70S6k is also common practice. Also, previous studies investigating a ketogenic diet confirmed that total expression of S6K is relatively stable in comparison to alterations in the phosphorylated form (McDaniel et al., 2011; Roberts et al., 2017; Bornstein et al., 2022).

- 1. **Metabolomics**
     1. **Determination of urinary creatinine concentration**

Spectrophotometry was used to measure urinary creatinine via the Jaffe reaction method, which was adapted from the manufacturing protocol of the QuantiChrom™ Creatinine Assay Kit (DICT-500, BioAssay Systems, USA). In this assay, a reaction takes place between creatinine and picric acid, while in an alkaline solution forming a yellow-orange adduct, of which the increase in absorbance is measured at wavelength of 510 nm*.* 10 µl of urine was mixed with a 200 µl working solution consisting of 0.1 M sodium hydroxide (NaOH), 0.3 mM ethylenediaminetetraacetic acid (EDTA); 0.1% (v/v) DMSO; 0.0004% (v/v) Tween^®^20*,* and 3 mM picric acid The assay was done in triplicate using a 96-well plate. Consequently, samples were then analysed using a Synergy™ HT Multi-detection microplate reader by measuring the linearity of the increase in absorbance at 510 nm for 5 min, in 1 min intervals. Lastly, absolute quantities were then calculated using a standard curve that ranged from 0-50 mg%. All urine samples used in multi-platform metabolomic assays creatinine concentrations were determined on two 96-well plates.

- - 1. **Quality Control Samples**

Urinary quality control (QC) samples were prepared for each mice group (ND, ND + Alc., KD and KD + Alc.) by pooling equal amounts of the relevant groups’ sample extracts together*.* Afterwards, the QC samples were aliquoted, stored and prepared by the same means as the other samples for each analytical platform respectively*.* QC samples were analysed in the beginning, after every 6 (GC-TOF-MS) or 5 (LC-MS/MS) samples, and at the end of each batch, for both GC-TOF-MS and LC-MS/MS analyses. Also, an in-house standard mixture (which contained all the metabolites to be analysed) including a QC sample spiked with this mixture, was analysed at the end of each batch during LC-MS/MS analysis.

- - 1. **LC-MS/MS sample preparation and analysis**

For LC-MS/MS sample preparation, a pre-planned volume (20 μl to 250 μl) of urine containing 0.0625 μmole creatinine, as well as internal standards and isotopes were evaporated under a stream of nitrogen gas at 37 °C*.* For sample derivatization, N-butanol:acetyl chloride (~300 μL) was added to each sample and which were then incubated at 50 °C for 1 h. Afterward, the butylated samples were dried under a stream of nitrogen gas at 37 °C. Lastly, the dried samples were reconstituted in a final volume of water:acetonitrile (50:50) (v/v) consisting of 0.1 % formic acid (50 μL). Following this, the samples were then thoroughly vortexed to dissolve the dried compounds. The final volume of the

|  |  |  |  |  |  |  |
| --- | --- | --- | --- | --- | --- | --- |

prepped urine samples were then transferred to 250 μL pulled point glass inserts*.* Finally, all the sample vials were loaded onto an Agilent© 1200 series auto sampler for LC-MS/MS analysis*.* LC-MS/MS analysis was performed as described by Mels et al. (2011) with slight adjustments. For LC-MS/MS analyses of amino acid-, carnitine- and acylcarnitine butyl esters a 1200 series liquid chromatograph coupled to a 6410-triple quadrupole-mass spectrometer (Agilent Technologies, USA) was used. The chromatographic separation was accomplished by injecting samples (1μl) on a C18 Zorbax SB-Aq (150 mm x 2.1 mm x 1.8 μm) reverse phase column (Agilent Technologies), kept at 35°C, with a flow rate of 0.2 ml/min during the entire analysis run. Additionally, a guard column was also attached to the column to prevent entry of particulate matter. Furthermore, the mobile phases, which consisted of water with 0.1% formic acid (mobile phase A) and acetonitrile with 0.1% formic acid (mobile phase B) were used. The chromatographic gradient started at a 5% mobile phase B and was maintained for 1 min, it was then followed with an increase to 23% of mobile phase B over a duration of 7 min. In addition, an isocratic hold of around 4 min was maintained at a 23% mobile phase B. Subsequently, mobile phase B was increased to 100% at 15 min and was kept there for 5 min. Lastly, the gradient declined to a 5% mobile phase B after 1 min and was maintained for 7 min (post-run) to guarantee that the column is in equilibrium before proceeding to the next analysis. The effluent coming from the liquid chromatograph was then diverted to the mass spectrometer, which made use of the positive electrospray ionisation (ESI) mode using the following source parameters: capillary voltage of 3 500 V; drying gas (nitrogen) at a flow rate of 7.5 l/min at 300°C; and nebuliser pressure of 30 psi. The spectra were acquired via the multiple reaction monitoring (MRM) mode with an electron multiplier voltage (EMV) of 300 V and a dwell time of 45 ms for all compounds. During the analysis, three-time windows were used. The first window diverted eluent prior to the detection of the first analyte (< 1.4 min)] away from the column and towards the waste to limit the exposure of the mass spectrometer to salts in the sample. The MRMs of all the butyl esters were distributed among the final two-time windows, thereby ensuring sufficient data points per compound peak. Last of all, Agilent©’s MassHunter Workstation Software (v B02.01; Data acquisition for 6400 Series Triple Quadrupole) and MassHunter Optimizer software (v B02.01) were used for data acquisition (with MRM configuration settings), while data extraction was accomplished using Agilent©’s MassHunter Workstation software (v B06.00; Qualitative Analysis and Quantitative Analysis).

| \| **Compound** \| **Precursor ion (m/z)** \| **Product ion (m/z)** \| **Fragmentor  voltage (V)** \| **Collision energy (eV)** \| \| --- \| --- \| --- \| --- \| --- \| \| Asparagine \| 189.1 \| 144.1 \| 89 \| 8 \| \| Ornithine \| 189.2 \| 70.2 \| 74 \| 20 \| \| Histidine \| 212.1 \| 110.1 \| 89 \| 16 \| \| Taurine \| 126 \| 107.9 \| 93 \| 10 \| \| Cystine \| 353 \| 130 \| 118 \| 16 \| \| Glycine \| 132.1 \| 76.1 \| 65 \| 4 \| \| Serine \| 162.1 \| 60.1 \| 94 \| 12 \| \| Glutamine \| 203.1 \| 84.1 \| 89 \| 20 \| \| Lysine_d4 \| 203.2 \| 84.1 \| 89 \| 20 \| \| Threonine \| 176.1 \| 74.1 \| 94 \| 12 \| \| β-Alanine \| 146.2 \| 72.1 \| 90 \| 10 \| \| Alanine \| 146.1 \| 44.2 \| 80 \| 15 \| \| Hydroxy-Proline \| 188.1 \| 68.1 \| 120 \| 35 \| \| Citrulline \| 232.2 \| 70.1 \| 89 \| 32 \| \| γ-Aminobutyric acid \| 160.1 \| 87.1 \| 79 \| 8 \| \| Carnitine-(methyl-d3) \| 221.2 \| 103.1 \| 127 \| 16 \| \| Carnitine \| 218.2 \| 103 \| 132 \| 16 \| \| Proline \| 172.1 \| 70.2 \| 94 \| 20 \| \| Creatine \| 188.1 \| 90.1 \| 104 \| 16 \| \| Arginine \| 231.2 \| 70.1 \| 103 \| 40 \| \| Valine_d8 \| 182.2 \| 80.2 \| 103 \| 12 \| \| Valine \| 174.2 \| 72.2 \| 89 \| 12 \| \| Acetylcarnitine \| 260.2 \| 85.1 \| 122 \| 24 \| \| Cystathionine \| 335.2 \| 190.1 \| 127 \| 16 \| \| Methionine \| 206.1 \| 104.1 \| 94 \| 8 \| \| Tyrosine \| 238.2 \| 136.1 \| 94 \| 12 \| \| 3-OH-Kynurenine \| 265.4 \| 136 \| 120 \| 10 \| \| Isoleucine_d10 \| 198.2 \| 96.2 \| 108 \| 12 \| \| Isoleucine \| 188.2 \| 86.2 \| 89 \| 8 \| \| Leucine \| 188.2 \| 86.2 \| 89 \| 8 \| \| Homocystine \| 381.2 \| 192.1 \| 95 \| 8 \| \| Phenylalanine_(ring-d5) \| 227.2 \| 125.1 \| 108 \| 16 \| \| Phenylalanine \| 222.2 \| 120.1 \| 108 \| 16 \| \| Aspartic acid \| 246.2 \| 144.1 \| 98 \| 12 \| \| Glutamic acid \| 260.2 \| 84.1 \| 89 \| 24 \| \| Tryptophan \| 261.2 \| 244.1 \| 94 \| 8 \| \| ButyryCarnitine \| 288.2 \| 85.1 \| 110 \| 20 \| \| Decanoylcarnitine \| 372.3 \| 85.1 \| 125 \| 28 \| \| Cysteine \| 178.1 \| 76 \| 180 \| 20 \| \| Hexanoycarnitine \| 316.2 \| 85.1 \| 137 \| 24 \| \| DodecanoyCarnitine \| 400.3 \| 85.1 \| 155 \| 28 \| \| Homocysteine \| 192 \| 90 \| 161 \| 12 \| \| TetradecanoyCarnitine \| 428.4 \| 85.1 \| 150 \| 28 \| \| OctanoyCarnitine-d3 \| 347.3 \| 85.1 \| 136 \| 24 \| \| OctanoyCarnitine \| 344.3 \| 85.1 \| 115 \| 28 \| \| IsovaleryCarnitine \| 302.2 \| 85.1 \| 105 \| 24 \| \| PalmitoyCarnitine \| 456.4 \| 85.1 \| 160 \| 36 \| \| OctadecanoyCarnitine \| 484 \| 85.1 \| 155 \| 36 \| \| OctadecanoyCarnitine-d3 \| 487.5 \| 85.1 \| 160 \| 36 \| |
| --- | --- | --- | --- | --- | --- | --- | --- | --- | --- | --- | --- | --- | --- | --- | --- | --- | --- | --- | --- | --- | --- | --- | --- | --- | --- | --- | --- | --- | --- | --- | --- | --- | --- | --- | --- | --- | --- | --- | --- | --- | --- | --- | --- | --- | --- | --- | --- | --- | --- | --- | --- | --- | --- | --- | --- | --- | --- | --- | --- | --- | --- | --- | --- | --- | --- | --- | --- | --- | --- | --- | --- | --- | --- | --- | --- | --- | --- | --- | --- | --- | --- | --- | --- | --- | --- | --- | --- | --- | --- | --- | --- | --- | --- | --- | --- | --- | --- | --- | --- | --- | --- | --- | --- | --- | --- | --- | --- | --- | --- | --- | --- | --- | --- | --- | --- | --- | --- | --- | --- | --- | --- | --- | --- | --- | --- | --- | --- | --- | --- | --- | --- | --- | --- | --- | --- | --- | --- | --- | --- | --- | --- | --- | --- | --- | --- | --- | --- | --- | --- | --- | --- | --- | --- | --- | --- | --- | --- | --- | --- | --- | --- | --- | --- | --- | --- | --- | --- | --- | --- | --- | --- | --- | --- | --- | --- | --- | --- | --- | --- | --- | --- | --- | --- | --- | --- | --- | --- | --- | --- | --- | --- | --- | --- | --- | --- | --- | --- | --- | --- | --- | --- | --- | --- | --- | --- | --- | --- | --- | --- | --- | --- | --- | --- | --- | --- | --- | --- | --- | --- | --- | --- | --- | --- | --- | --- | --- | --- | --- | --- | --- | --- | --- | --- | --- | --- | --- | --- | --- | --- | --- | --- | --- | --- | --- | --- | --- | --- | --- | --- | --- |

**Table S2.** MRM parameters for metabolites and internal standard isotopes monitored.

- - 1. **GC-TOF-MS sample preparation and analysis**

For GC-TOF-MS sample preparation (20 μl to 250 μl) of urine containing 0.0625 μmole creatinine, as well as internal standards dried under a stream of nitrogen gas at 37°C. Derivatization of the samples was accomplished through oximation and silylation. For oximation, methoxyamine hydrochloride (20 mg/mL in pyridine, 50 μL) was added and the samples were subsequently vortexed for 1 min to dissolve the dried compounds*.* Afterwards, the samples were then incubated at 60 °C for 1 hour*.* Next, the samples were silylated by adding BSTFA (50 μL) containing 1 % (v/v) TMCS and incubated at 60 °C for 1 hour. The final sample volume (100 μL) for each sample was then transferred to 250 μL pulled point glass inserts. Finally, each vial was loaded onto via a 7890A gas chromatograph (Agilent Technologies, USA) with an Agilent© 7693 autosampler coupled to a LECO Pegasus HT time-of-flight mass spectrometer with an electron impact (EI) ionization source.

The GC-TOF-MS protocol described by Lindeque et al. (2013) was used for this study. The samples (1 µL) were then injected using the spitless mode *and* a Phenomenex Zebron GC FocusLiner® Inlet Liner for Agilent (split/splitless, single taper w/wool, 4 mm ID x 78.5 mm L x 6.3 mm OD) was fitted on the GC system*.* Throughout the entire analysis, the front inlet’s temperature was kept at 250 °C and the carrier gas (helium) was kept at a constant flow rate of 1.5 mL/min. A Restek Rxi-5Sil MS (30m x 250 µm x 0.25 µm) column was used for chromatography analysis and the initial GC oven temperature was held at 40 °C for 1 min. Afterwards, the oven temperature was then increased by 7 °C/min until reaching 120 °C, followed by 10 °C/min until reaching 230 °C and finally 13 °C/min until it reached 300 °C*,* which was then maintained at that temperature for 2.5 min before being cooled to 40 °C with a total run time of ~ 35 min*.* During the entire analysis, the transfer line and ion source temperatures were respectively maintained at 225 °C and 200 °C. For the detection of metabolites, mass spectrometry was operated using electron impact (EI) ionization (- 70 V) mode which allowed for the fragmentation of all eluting compounds*.* Using a solvent delay of 480 seconds, data was acquired at a rate of 20 spectra/second with a mass-to-charge (m/z) ratio scan range of 50–950 amu with a detector voltage of ~1 500 V. Peaks were detected by 5 apexing masses using a peak width of 3 seconds and a signal-to-noise (S/N) ratio of > 20. For data acquisition and extraction, the LECO Corporation ChromaTOF software (v 4.5x) was used*.* The software’s Statistical Compare feature was used to align peaks, by automatic baseline removal via the “spanning” tracking method (offset of 1; just above the noise) as well as auto smoothing. Spectral matching was carried making use of the NIST11 commercial- as well as an in-house mass spectral library to identify important analytes. For metabolite identification, an identification confidence level was ascribed to each feature as described by Schymanski et al. (2014). All the features that were unidentified were excluded from the dataset*.* Features were considered as a Level 3 if a tentative structure was obtained (based on either matching intact molecular mass or fragmentation spectra). In contrast, Level 2 features had greater structure identity based on additional evidence. For features labelled as Level 1, the structure was identified with the spectral and retention time corresponding with a reference standard.

- - 1. **Measuring inflammatory biomarkers in mice serum**

The concentration levels of pro-inflammatory biomarkers of IL6, IL10, MCP1, TNF, IFN-γ and IL12 were measured using the BD Cytometric bead array (CBA) mouse inflammation kit (BD Biosciences, cat# 552364) according to the protocol of the kit, which made use of a multiplex bead array assay coupled with flow cytometry. The samples as well as standards were analysed via a BD Accuri C6 flow cytometer. Afterwards, standard curves were produced and then regression analysis was applied using FlowJo (version 10.8) and FCS Express software (De Novo, version 6) to determine the concentrations of the biomarkers (in pg/ml) of the mice serum samples.

- - 1. **Data processing and normalization**

For data processing and normalisation, in-house protocols were used along with the relevant literature recommendations. The stages that were followed are summarised in Fig. S1 and include: 1) The removal of inconsistent features; 2) Elimination of contaminant peaks; 3) Removal of inconsistent samples; 4) The use of internal standards for post-analysis normalisation, to minimize systematic and technical variations; 5) Removal of inconsistent features making use of QC samples; and 6*)* Missing value imputation (MVI) to replace missing values with a non-zero value (while maintaining the data structure)*.* Following this, data was transformed (glog) to improve the data set normality for greater comparability of metabolite intensities between and within samples before applying statistical analysis*.* Lastly, the removal of outlier samples was also done with the data sets.


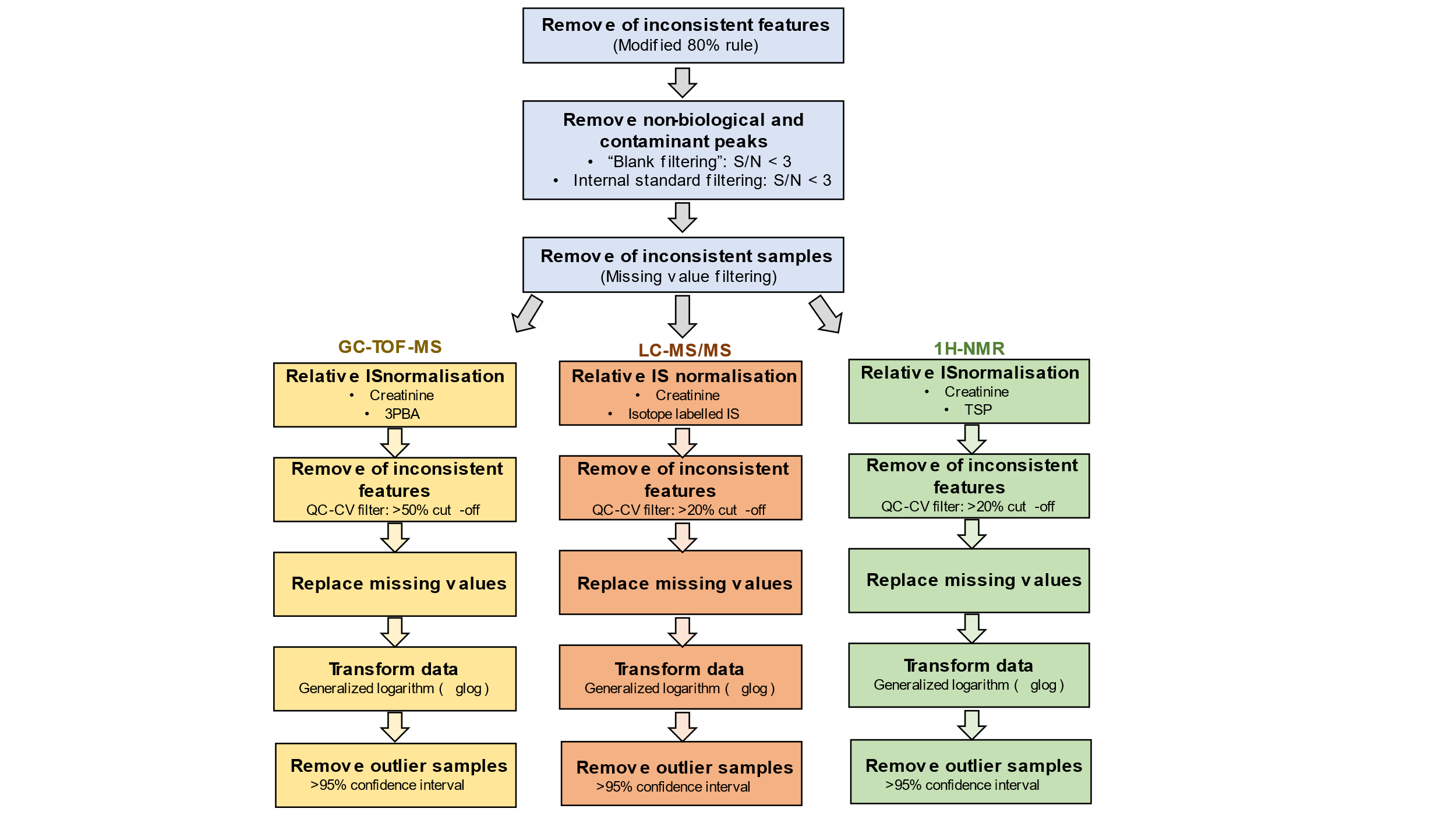


**Fig. S1.** Data processing and normalization of extracted metabolomics data sets. The data processing stages involved the removal of inconsistent and contaminant peaks, removal of inconsistent samples, following analysis.

1. **Results**

| **A B**  **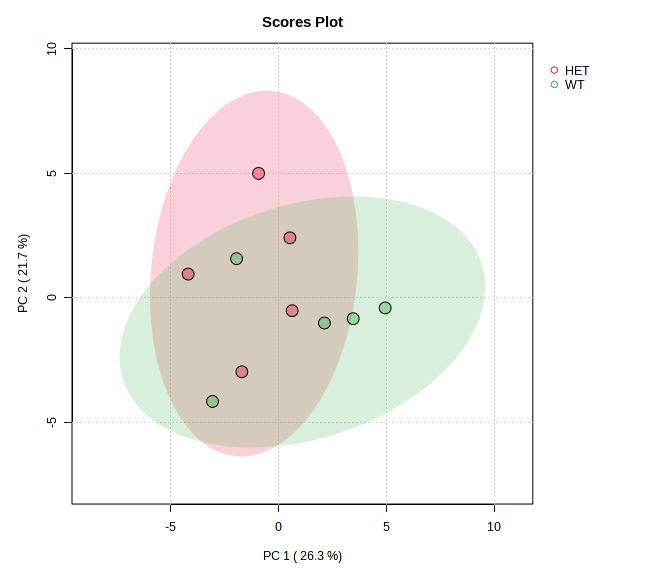 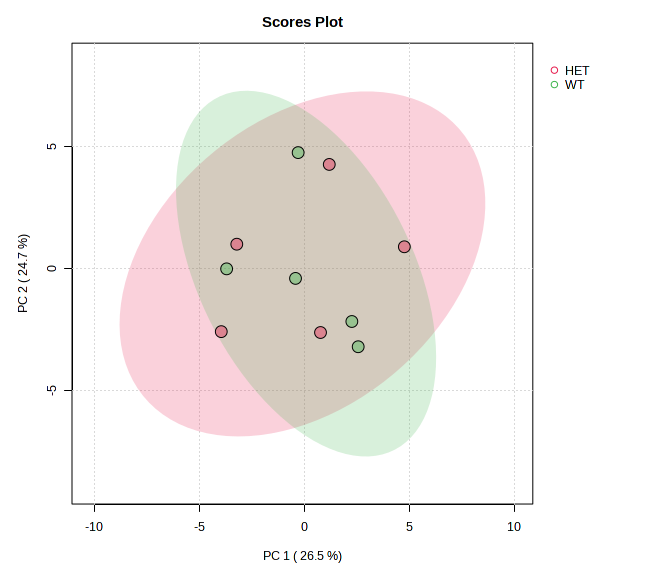**  **C D**  **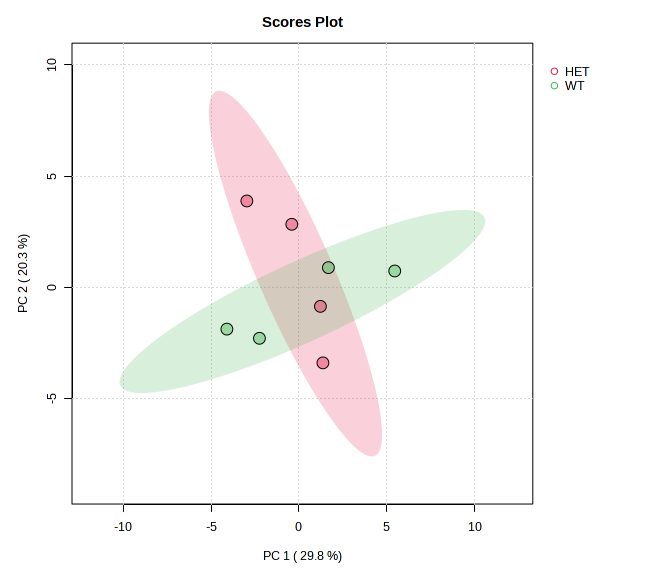 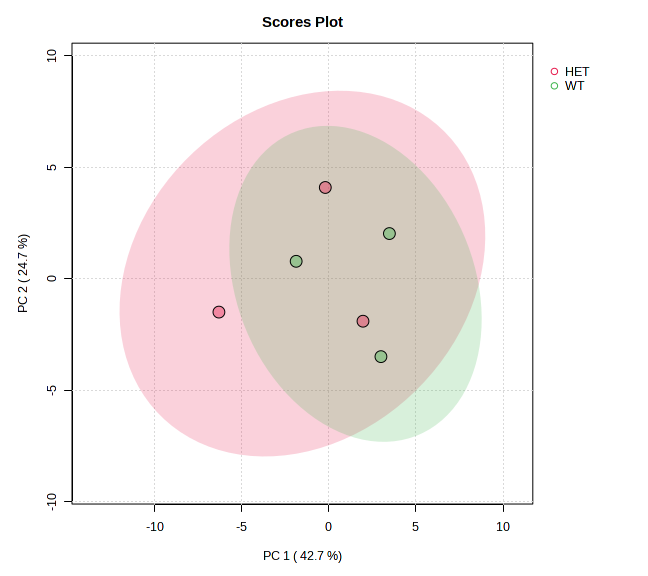** |
| --- |

**Fig. S2.** PCA scores plot illustrates no natural separation between (A) WT (n=5) and HET mice (n=5) on a normal diet, (B) WT (n=5) and HET mice (n=5) on a normal diet with alcohol consumption, (C) WT (n=4) and HET (n=4) mice on a ketogenic diet and (D) WT (n=3) and HET (n=3) mice on a ketogenic diet with alcohol consumption, when the different genotypes (n=6 to 10 per group) were compared. Each metabolite was normalised to its respective internal standard (based on the relative metabolomics platform) and subsequently log transformed, as well as auto-scaled using MetaboAnalyst 5.0.

| **KD vs ND** | | | | | | | | | | | |
| --- | --- | --- | --- | --- | --- | --- | --- | --- | --- | --- | --- |
| **Compound** | ***p*-value*** | ***d*-value** | **↑/↓** | **ID Level & Class** | **Platform** | **Compound** | ***p*-value*** | ***d*-value** | **↑/↓** | **ID Level**  **& Class** | **Platform** |
| 2-Hydroxyadipic acid | p<0.001 | 1.45 | ↑ | 3; O | GC-TOF-MS | 2-Hydroxyisobutyric acid | p<0.001 | 6.12 | *↓* | 3; O | GC-TOF-MS |
| 2-Ketoisocaproic acid | p<0.001 | 2.17 | ↓ | 2; O | ^1^H-NMR | 2-Keto-3-methylvaleric acid | p<0.001 | 1.72 | ↓ | 3; O | GC-TOF-MS |
| 2-Methylbutyrylglycine | p<0.001 | 1.64 | ↑ | 3; O | GC-TOF-MS | 5-Hydroxyindole-3-acetic acid | p<0.001 | 5.04 | *↓* | 3 | GC-TOF-MS |
| 3-Ureidopropionic acid | 0.004 | 1.46 | ↓ | 2; O | ^1^H-NMR | Butyryl-L-Carnitine | p<0.001 | 1.13 | ↑ | 1; A, F | LC-MS/MS |
| 4-Ketoglucose | p<0.001 | 2.88 | *↓* | 3; O | GC-TOF-MS | Cystathionine | 0.010 | 1.10 | ↓ | 1; A | LC-MS/MS |
| Alanine | p<0.001 | 3.51 | ↓ | 1; A | LC-MS/MS | Cystine | 0.001 | 1.80 | ↓ | 1; A | LC-MS/MS |
| Allantoin | p<0.001 | 1.63 | ↓ | 2; O | ^1^H-NMR | D-Arabino-Hexonic acid | 0.008 | 4.57 | *↓* | 3; O | GC-TOF-MS |
| Arginine | p<0.001 | 2.58 | ↓ | 1; A | LC-MS/MS | D-Gluconic acid | 0.047 | 0.84 | ↑ | 3; O | GC-TOF-MS |
| b-Aminoisobutyric acid | 0.048 | 1.18 | ↑ | 3; O | GC-TOF-MS | Dimethylamine | p<0.001 | 1.89 | ↓ | 2; O | ^1^H-NMR |
| Betaine | p<0.001 | 4.30 | ↓ | 2; O | ^1^H-NMR | Ethanolamine | p<0.001 | 4.53 | ↓ | 2; O | ^1^H-NMR |
| Citric acid | p<0.001 | 3.02 | ↓ | 2; T | ^1^H-NMR | Fumaric acid | 0.006 | 1.70 | ↓ | 3; T | GC-TOF-MS |
| Dodecanoyl-L-Carnitine | p<0.001 | 4.37 | ↑ | 1; A, F | LC-MS/MS | GABA | p<0.001 | 2.97 | ↓ | 1; A | LC-MS/MS |
| Hydroquinone | 0.029 | 5.43 | *↓* | 3; O | GC-TOF-MS | Glutaric acid | p<0.001 | 15.66 | *↓* | 3; O | GC-TOF-MS |
| Hypoxanthine | 0.035 | 6.02 | *↓* | 3; O | GC-TOF-MS | Homocysteine | p<0.001 | 1.73 | ↓ | 1; A | LC-MS/MS |
| Inositol | p<0.001 | 8.67 | *↓* | 3; O | GC-TOF-MS | Isocitric acid | p<0.001 | 5.86 | ↓ | 3; T | GC-TOF-MS |
| L-Carnitine | p<0.001 | 2.06 | ↓ | 1; A, F | LC-MS/MS | Isoleucine | 0.026 | 1.59 | ↓ | 1; A | LC-MS/MS |
| Myo-Inositol | p<0.001 | 10.45 | *↓* | 3; O | GC-TOF-MS | Leucine | 0.049 | 1.47 | ↓ | 1; A | LC-MS/MS |
| N-Valerylglycine | 0.001 | 3.53 | *↓* | 3; O | GC-TOF-MS | Gulonic acid | 0.021 | 1.06 | ↑ | 3; O | GC-TOF-MS |
| Oxalic acid | 0.004 | 1.01 | ↑ | 3; O | GC-TOF-MS | Malic acid | p<0.001 | 1.87 | ↓ | 3; T | GC-TOF-MS |
| Palmitoyl-L-Carnitine | 0.040 | 1.97 | ↓ | 1; A, F | LC-MS/MS | Methionine | 0.002 | 1.78 | ↓ | 1; A | LC-MS/MS |
| Phenylacetylglycine | p<0.001 | 1.28 | ↓ | 2; O | ^1^H-NMR | Methylamine | p<0.001 | 0.00 | ↓ | 2; O | ^1^H-NMR |
| Succinic acid | p<0.001 | 3.46 | ↓ | 2; T | ^1^H-NMR | Serine | p<0.001 | 2.71 | ↓ | 1; A | LC-MS/MS |
| Valeric acid | p<0.001 | 4.93 | *↓* | 3; O | GC-TOF-MS | Tricarballylic acid | p<0.001 | 7.09 | *↓* | 3; O | GC-TOF-MS |
| α-Ketoglutaric acid | p<0.001 | 1.83 | ↓ | 2; O | ^1^H-NMR | Trimethylamine | p<0.001 | 1.65 | ↓ | 2; O | ^1^H-NMR |
| β-Alanine | p<0.001 | 3.51 | ↓ | 1; A | LC-MS/MS |  |  |  |  |  |  |
| **KD+Alc vs ND** | | | | | | | | | | | |
| **Compound** | ***p*-value*** | ***d*-value** | **↑/↓** | **ID Level & Class** | **Platform** | **Compound** | ***p*-value*** | ***d*-value** | **↑/↓** | **ID Level & Class** | **Platform** |
| 2-Hydroxyisobutyric acid | 0.003 | 1.73 | *↓* | 3; O | GC-TOF-MS | 2-Hydroxyadipic acid | p<0.001 | 8.18 | ↑ | 3; O | GC-TOF-MS |
| 2-Hydroxyvaleric acid | 0.002 | 3.73 | ↑ | 3; O | GC-TOF-MS | 2-Keto-3-methylvaleric acid | 0.001 | 1.58 | *↓* | 3; O | GC-TOF-MS |
| 2-Ketoisocaproic acid | p<0.001 | 6.40 | ↓ | 2; O | ^1^H-NMR | 3-Hydroxyadipic acid | p<0.001 | 4.73 | ↑ | 3; O | GC-TOF-MS |
| 2-Methylbutyrylglycine | p<0.001 | 7.59 | ↑ | 3; O | GC-TOF-MS | 4-Ketoglucose | p<0.001 | 1.68 | *↓* | 3; O | GC-TOF-MS |
| 2-Pyrrolidone-5-carboxylic acid | 0.012 | 2.90 | ↑ | 3; O | GC-TOF-MS | Butyryl-L-Carnitine | 0.004 | 2.81 | ↑ | 1; A, F | LC-MS/MS |
| 3-Hydroxybutyrate | 0.022 | 606.94 | ↑ | 2; O | ^1^H-NMR | Cystine | p<0.001 | 2.41 | ↓ | 1; A | LC-MS/MS |
| 5-Hydroxyindole-3-acetic acid | p<0.001 | 1.35 | *↓* | 3; O | GC-TOF-MS | Dimethylamine | p<0.001 | 370.17 | ↓ | 2; O | ^1^H-NMR |
| Alanine | p<0.001 | 3.16 | ↓ | 1; A | LC-MS/MS | Ethanolamine | p<0.001 | 1027.71 | ↓ | 2; O | ^1^H-NMR |
| Allantoin | p<0.001 | 9.79 | ↓ | 2; O | ^1^H-NMR | Fumaric acid | p<0.001 | 1.42 | *↓* | 3; T | GC-TOF-MS |
| Arginine | p<0.001 | 2.41 | ↓ | 1; A | LC-MS/MS | GABA | p<0.001 | 2.89 | ↓ | 1; A | LC-MS/MS |
| Betaine | p<0.001 | 5.39 | ↓ | 2; O | ^1^H-NMR | Glutaric acid | p<0.001 | 2.60 | *↓* | 3; O | GC-TOF-MS |
| Citric acid | p<0.001 | 2.99 | ↓ | 2; T | ^1^H-NMR | Homocysteine | p<0.001 | 1.71 | ↓ | 1; A | LC-MS/MS |
| D-Arabino-Hexonic acid | 0.039 | 1.11 | *↓* | 3; O | GC-TOF-MS | Homocystine | p<0.001 | 1.07 | ↑ | 1; A | LC-MS/MS |
| D-Gluconic acid | p<0.001 | 5.11 | ↑ | 3; O | GC-TOF-MS | Inositol | p<0.001 | 1.96 | *↓* | 3; O | GC-TOF-MS |
| Dodecanoyl-L-Carnitine | p<0.001 | 3.58 | ↑ | 1; A, F | LC-MS/MS | Isocitric acid | p<0.001 | 2.55 | *↓* | 3; T | GC-TOF-MS |
| Glyceric acid | 0.005 | 1.56 | *↓* | 3; O | GC-TOF-MS | Isoleucine | 0.041 | 1.37 | ↓ | 1; A | LC-MS/MS |
| Glycine | 0.033 | 329.59 | ↓ | 2; A | ^1^H-NMR | Leucine | 0.050 | 1.33 | ↓ | 1; A | LC-MS/MS |
| L-Carnitine | p<0.001 | 1.99 | ↓ | 1; A, F | LC-MS/MS | Malic acid | p<0.001 | 1.71 | *↓* | 3; T | GC-TOF-MS |
| Gulonic acid | p<0.001 | 5.12 | ↑ | 3; O | GC-TOF-MS | Methionine | 0.0096 | 1.79 | ↓ | 1; A | LC-MS/MS |
| Phenylacetylglycine | p<0.001 | 12.81 | ↓ | 2; O | ^1^H-NMR | Methylamine | p<0.001 | 0.00 | ↓ | 2; O | ^1^H-NMR |
| Succinic acid | p<0.001 | 11.44 | ↓ | 2; T | ^1^H-NMR | Myo-Inositol | p<0.001 | 1.82 | *↓* | 3; O | GC-TOF-MS |
| Tricarballylic acid | 0.014 | 1.37 | *↓* | 3; O | GC-TOF-MS | Serine | 0.003 | 2.07 | ↓ | 1; A | LC-MS/MS |
| Valeric acid | p<0.001 | 2.26 | *↓* | 3; O | GC-TOF-MS | Trimethylamine | p<0.001 | 372.02 | ↓ | 2; O | ^1^H-NMR |
| β-Alanine | p<0.001 | 3.55 | ↓ | 1; A | LC-MS/MS |  |  |  |  |  |  |
| **ND+Alc vs ND** | | | | | | | | | | | |
| **Compound** | ***p*-value*** | ***d*-value** | **↑/↓** | **ID Level & Class** | **Platform** | **Compound** | ***p*-value*** | ***d*-value** | **↑/↓** | **ID Level & Class** | **Platform** |
| Acetic acid | 0.038 | 0.74 | ↑ | 2; O | ^1^H-NMR | 3-Methyladipic acid | 0.004 | 1.05 | *↓* | 3; O | GC-TOF-MS |
| Allantoin | 0.005 | 1.02 | ↓ | 2; O | ^1^H-NMR | Hypoxanthine | 0.003 | 1.02 | ↑ | 3; O | GC-TOF-MS |
| Arginine | 0.001 | 1.23 | ↑ | 1; A | LC-MS/MS | Isoleucine | 0.002 | 1.71 | ↑ | 1; A | LC-MS/MS |
| Dimethylamine | 0.005 | 1.07 | ↓ | 2; O | ^1^H-NMR | Leucine | 0.003 | 1.61 | ↑ | 1; A | LC-MS/MS |

**Table S3.** The significant discriminatory metabolites with an identification confidence Level of 1, 2 and 3 together with an FDR-corrected p-value <0.05 and d-value ≥0.8 between the different dietary groups, obtained via one-way ANOVA followed by a post-hoc Tukey test. Arrows indicate an increase (↑) or decrease (↓) relative to the metabolite levels of the control group. The identification (ID) level in conjunction with the relative metabolite class (amino acid, A, fatty acids, F; TCA cycle, T; or other, O) is listed for each metabolite. GABA, γ-aminobutyric acid. The relative platform used to identify each metabolites is also listed. GC-TOF-MS, gas chromatography time-of-flight mass spectrometry; LC-MS/MS, liquid chromatography with tandem mass spectrometry; ^1^H-NMR, hydrogen-1 nuclear magnetic resonance

| **Interactions between diet and alcohol** | | | |
| --- | --- | --- | --- |
| **Compound** | ***p*-value*** | **ID Level** | **Class** |
| Alloxanoic acid | p<0.001 | 3 | A |
| Creatine | 0.012 | 1 | O |
| Cysteine | 0.030 | 1 | A |
| Hydroxyproline | 0.042 | 1 | A |
| Isobutyric acid | 0.008 | 3 | A |
| Taurine | 0.045 | 2 | A |

**Table S4.** The significant discriminatory metabolites with an interaction between diet and alcohol. An identification confidence Level of 1, 2 and 3 is given, together with an FDR-corrected p-value <0.05 and d-value ≥0.8, obtained via a two-way ANOVA followed by a post-hoc Tukey test. Arrows indicate an increase (↑) or decrease (↓) relative to the metabolite levels of the control group. The identification (ID) level in conjunction with the relative metabolite class (amino acid, A or other, O) is listed for each metabolite.

| **A B**  **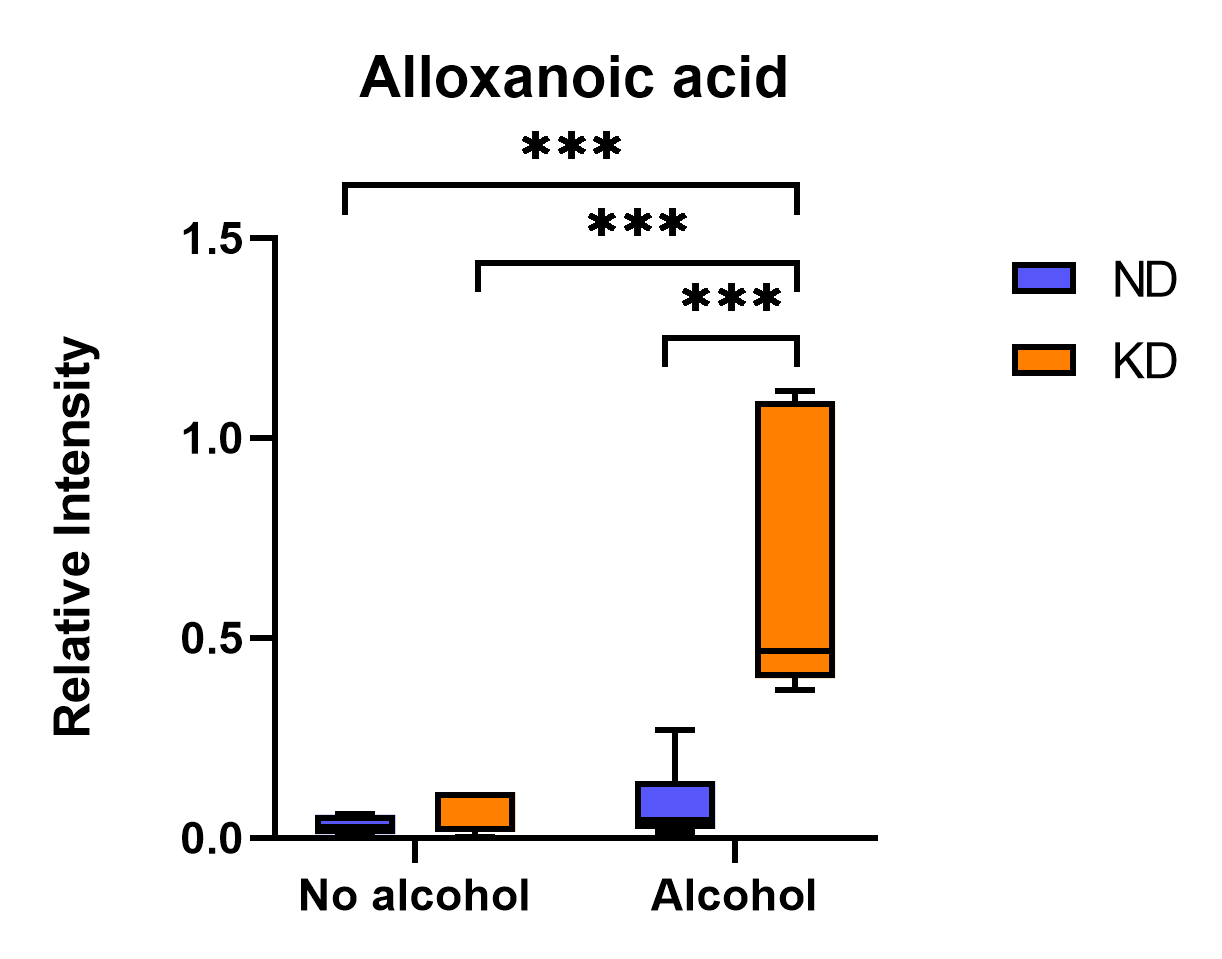 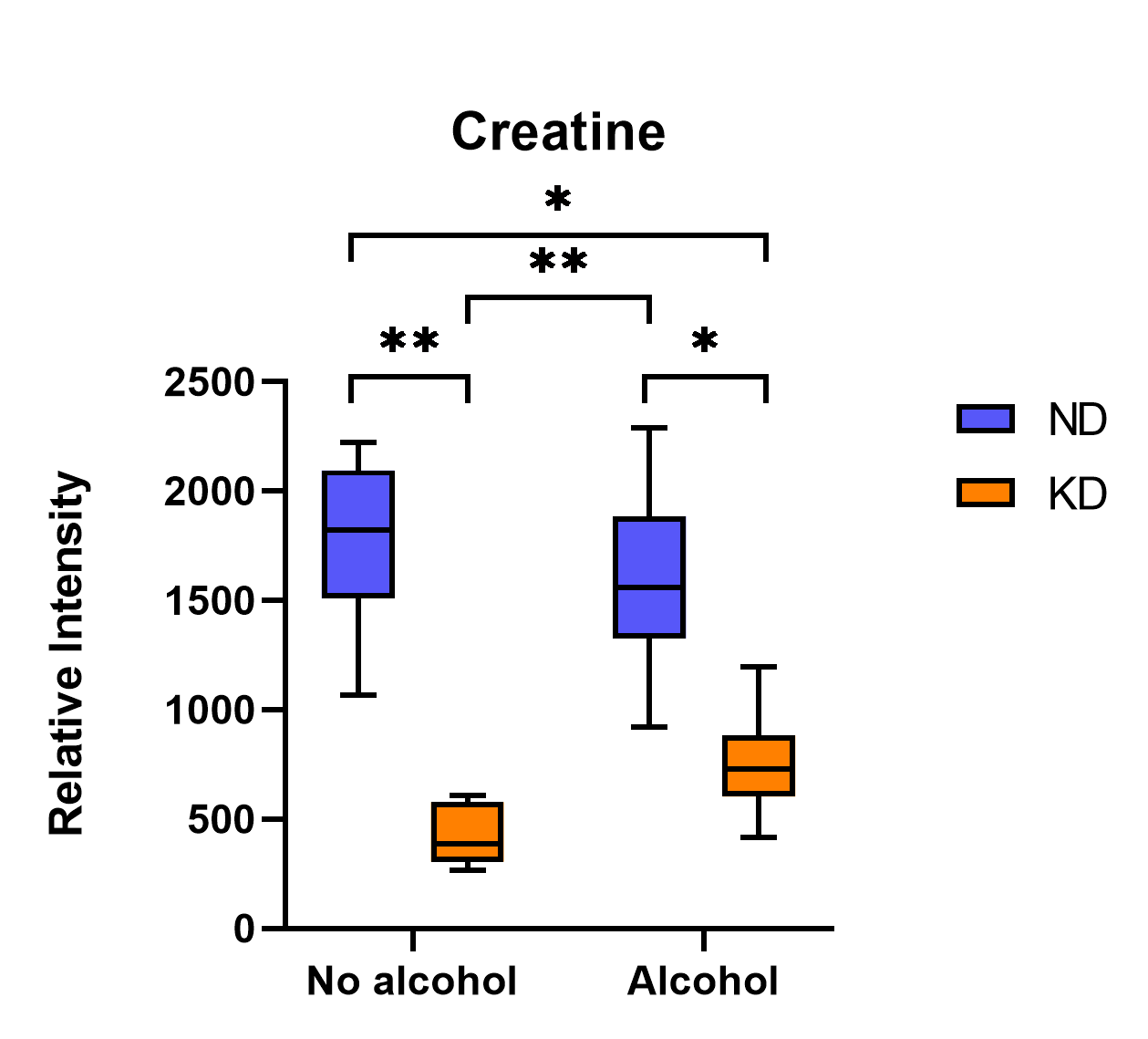**  **C D**  **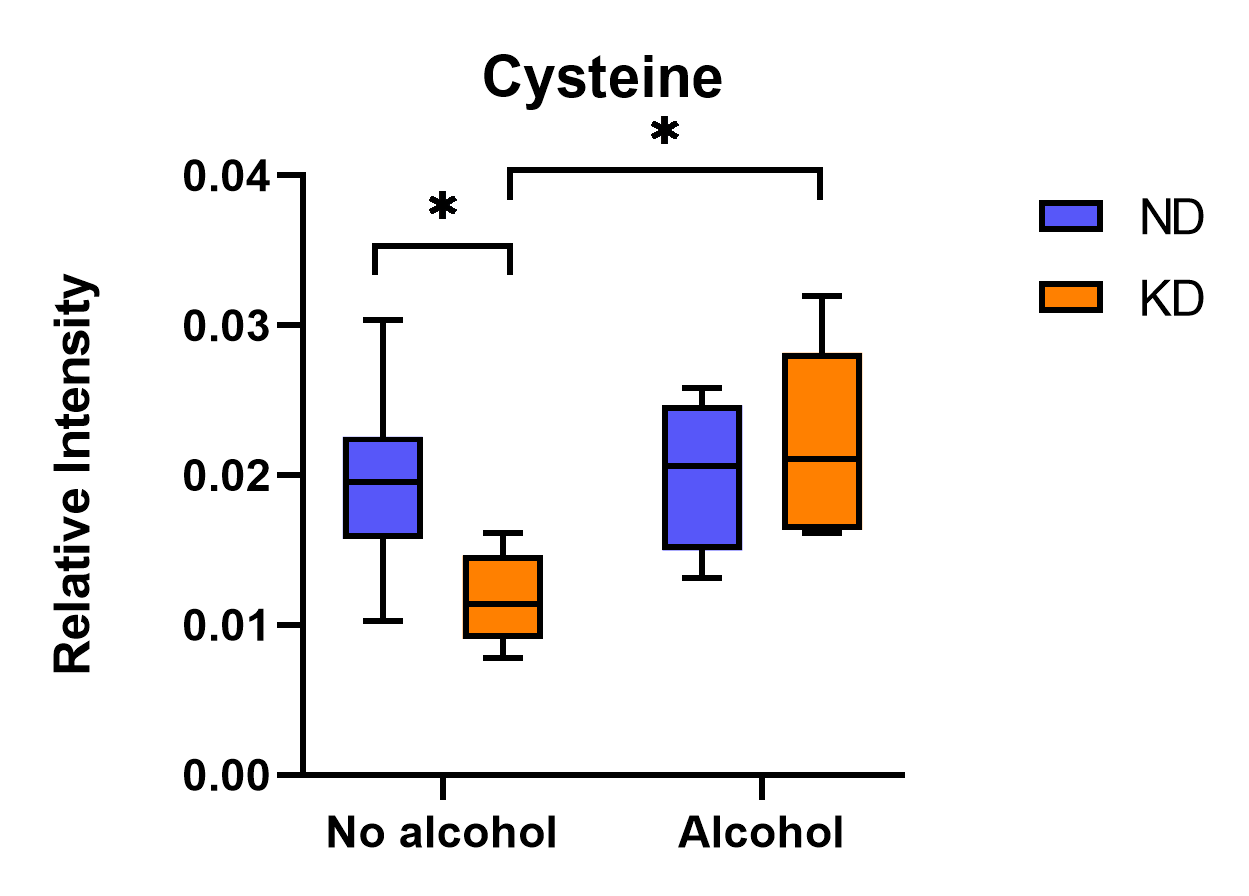 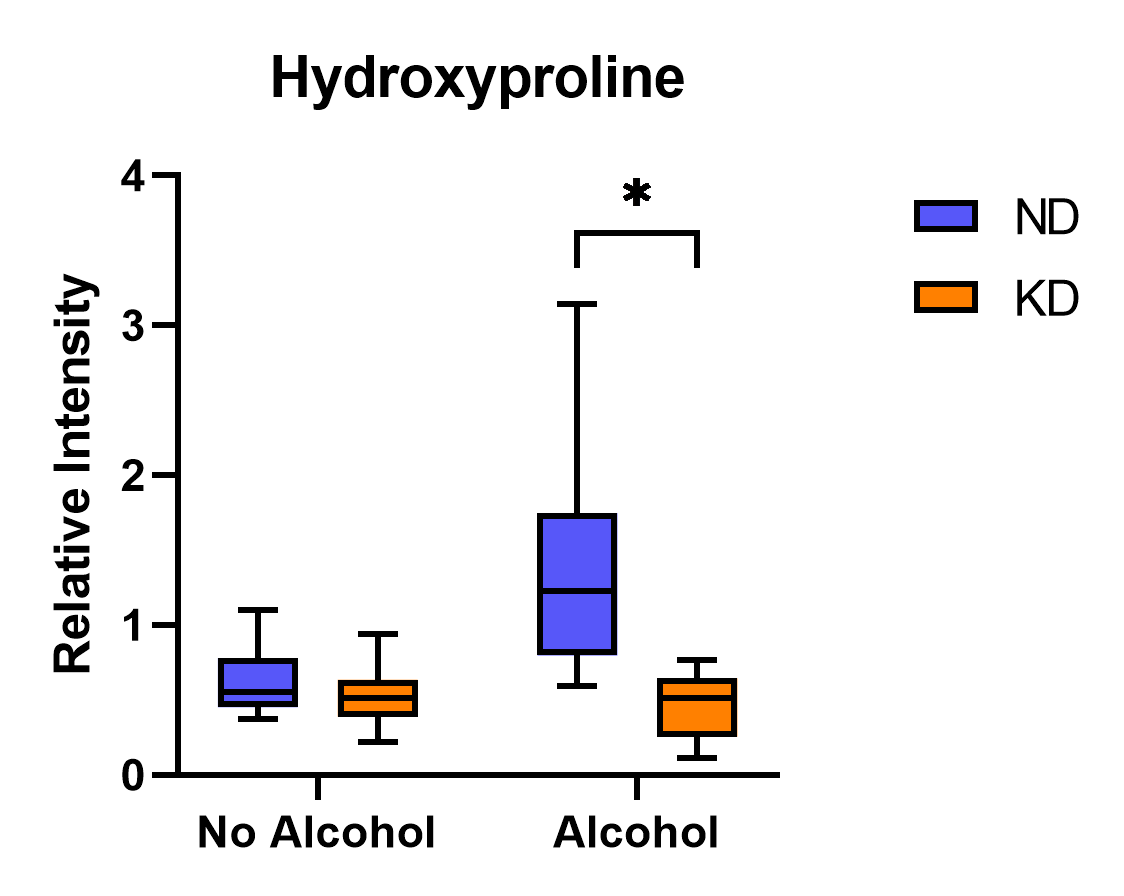**  **E F**  **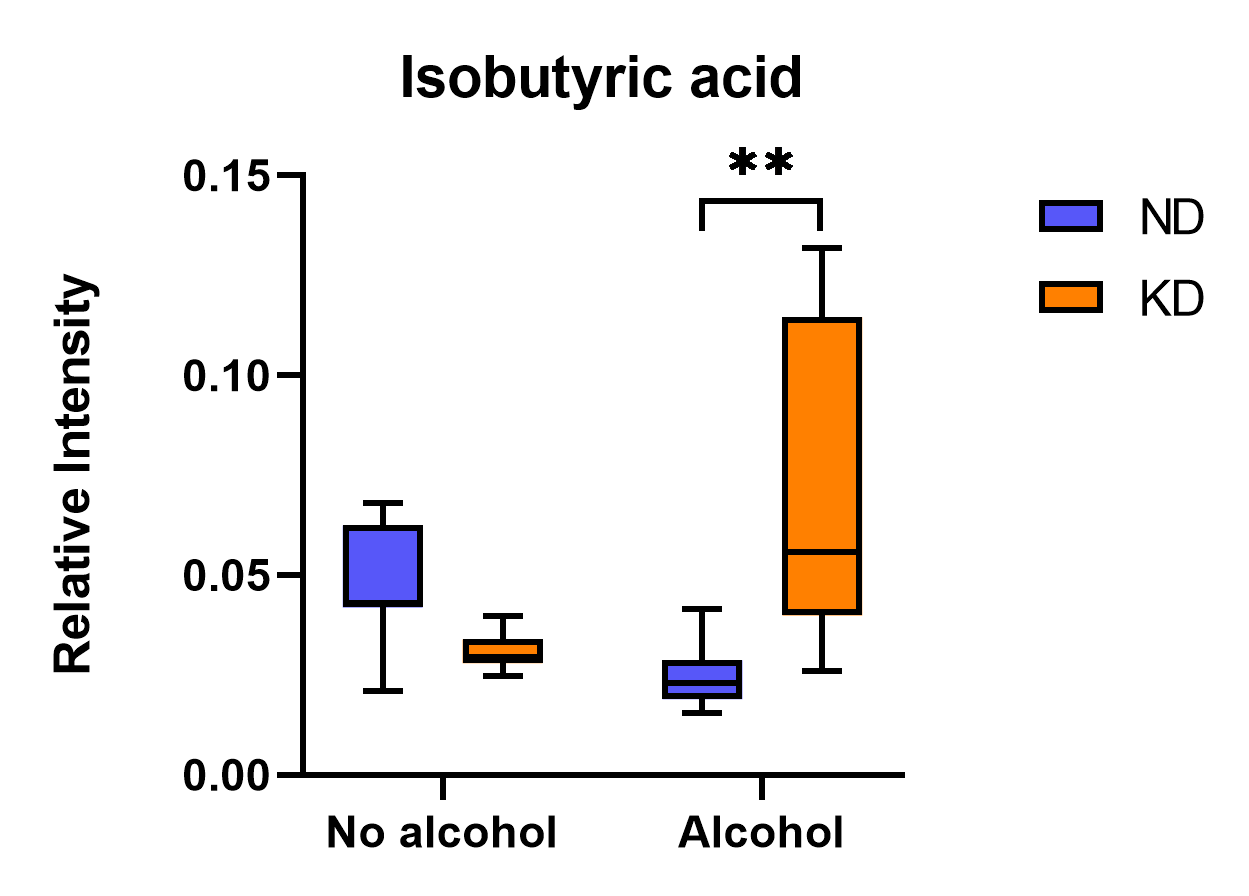 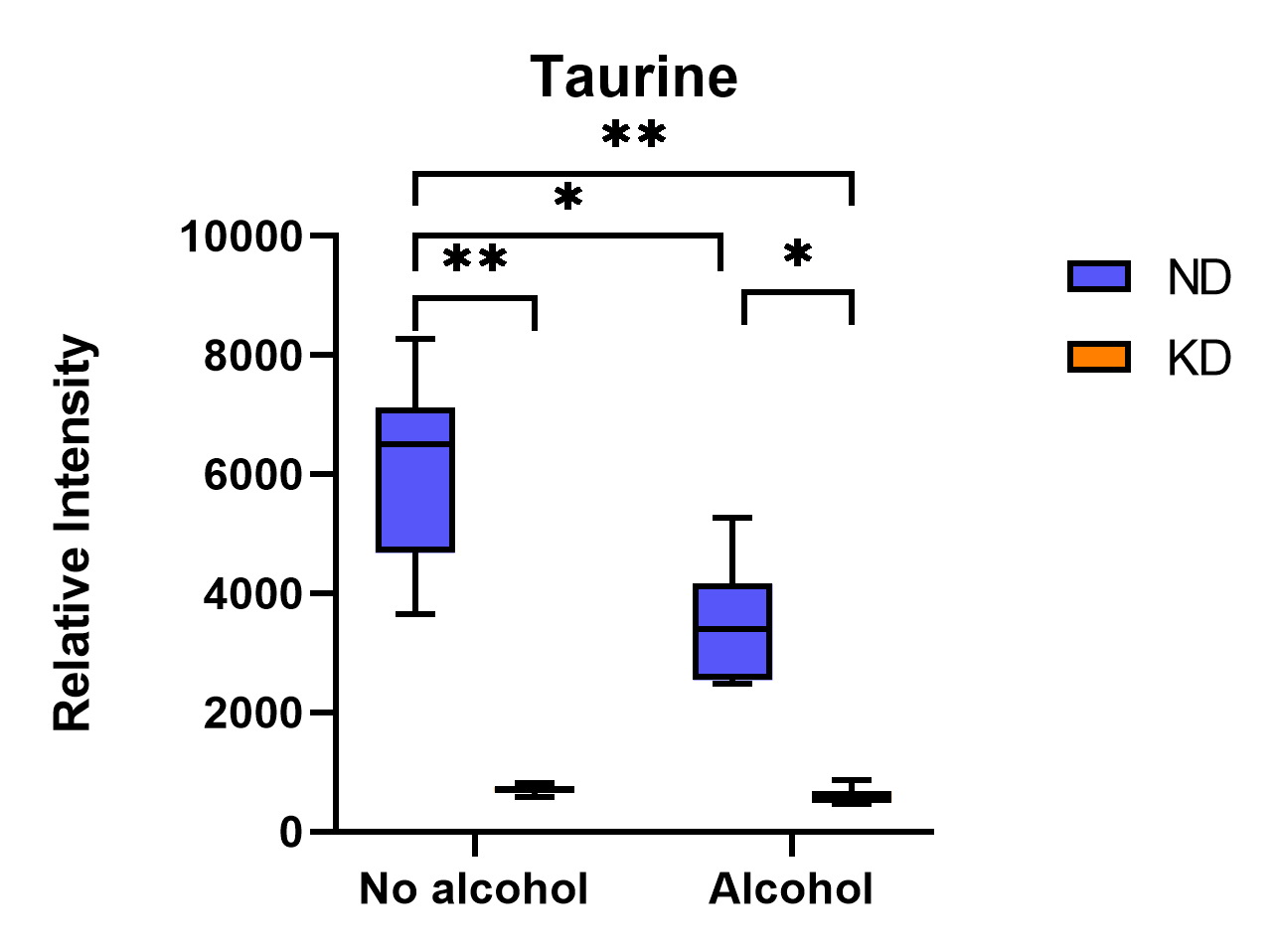** |
| --- |

**Fig. S3.** Box plots indicate the relative urinary levels of the significant metabolites with an interaction between diet and alcohol (n=6-10 per group). **p < 0.05, **p < 0.01* and ****p < 0.001*, obtained via a post-hoc Tukey’s test.


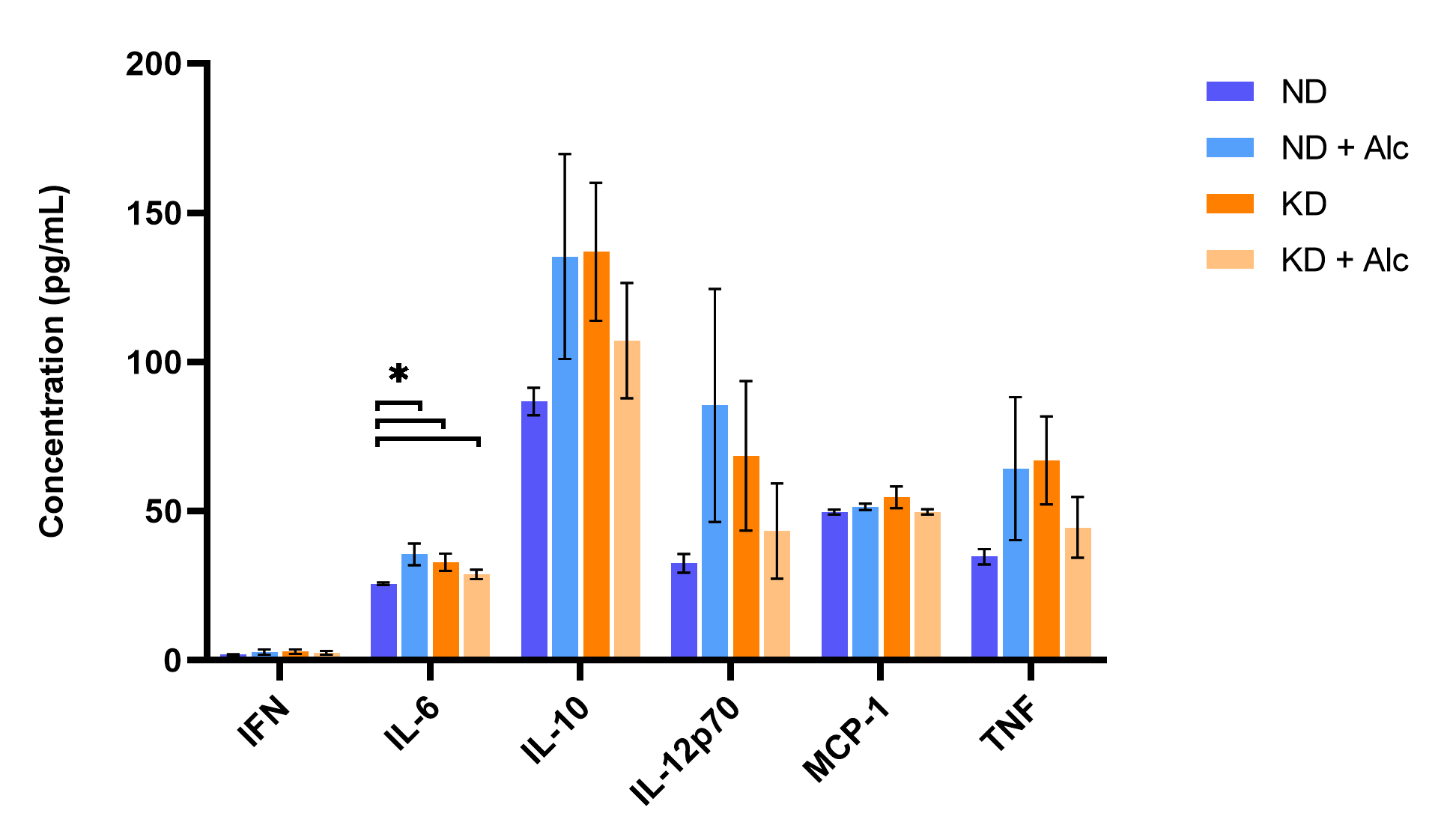


**Fig. S4.** The concentration levels (pg/mL) of pro-inflammatory biomarkers as measured in the serum of mice from all four dietary groups (n = 6 per group). The error bars represent the standard error. *p < 0.05 vs. the control group.

# References

Bornstein, R., James, K., Stokes, J., Park, K. Y., Kayser, E.-B., Snell, J., . . . Johnson, S. C. (2022). Differential effects of mTOR inhibition and dietary ketosis in a mouse model of subacute necrotizing encephalomyelopathy. Neurobiol Dis, 163, 105594. doi:10.1016/j.nbd.2021.105594

Chen, J., Zhang, P., Lv, M., Guo, H., Huang, Y., Zhang, Z., & Xu, F. (2017). Influences of Normalization Method on Biomarker Discovery in Gas Chromatography–Mass Spectrometry-Based Untargeted Metabolomics: What Should Be Considered? *Analytical Chemistry*, 89(10), 5342-5348. doi:10.1021/acs.analchem.6b05152 Di

Di Guida R, Engel J, Allwood JW, Weber RJ, Jones MR, Sommer U, Viant MR, Dunn WB. Non-targeted UHPLC-MS metabolomic data processing methods: a comparative investigation of normalisation, missing value imputation, transformation and scaling. *Metabolomics*. 2016;12:93. doi: 10.1007/s11306-016-1030-9.

Hsin, I. L., Shen, H. P., Chang, H. Y., Ko, J. L., & Wang, P. H. (2021). Suppression of PI3K/Akt/mTOR/c-Myc/mtp53 Positive Feedback Loop Induces Cell Cycle Arrest by Dual PI3K/mTOR Inhibitor PQR309 in Endometrial Cancer Cell Lines. Cells, 10(11). doi: doi.org/10.3390/cells10112916

Lindeque, J., Hidalgo, J., Louw, R., & Westhuizen, F. (2013). Systemic and organ specific metabolic variation in metallothionein knockout mice challenged with swimming exercise. *Metabolomics*, 9, 418-432. [doi:10.1007/s11306-012-0459-8](https://doi.org/10.1007/s11306-012-0459-8)

Lindeque, J. Z., Jansen van Rensburg, P. J., Louw, R., van der Westhuizen, F. H., Florit, S., Ramírez, L., . . . Hidalgo, J. (2015). Obesity and metabolomics: metallothioneins protect against high-fat diet-induced consequences in metallothionein knockout mice. *Omics*, 19(2), 92-103. doi: 10.1089/omi.2014.0087.

McDaniel, S., Rensing, N., Yamada, K., & Wong, M. (2011). The ketogenic diet inhibits the mammalian target of rapamycin (mTOR) pathway. Epilepsia, 52, e7-11. doi:10.1111/j.1528-1167.2011.02981.x

Mels, C. M., Jansen van Rensburg, P., van der Westhuizen, F. H., Pretorius, P. J., & Erasmus, E. (2011). Increased excretion of c4-carnitine species after a therapeutic acetylsalicylic Acid dose: evidence for an inhibitory effect on short-chain Fatty Acid metabolism. *ISRN* *Pharmacol*, 2011, 851870. doi: 10.5402/2011/851870.

Roberts, M. N., Wallace, M. A., Tomilov, A. A., Zhou, Z., Marcotte, G. R., Tran, D., . . . Lopez-Dominguez, J. A. (2017). A Ketogenic Diet Extends Longevity and Healthspan in Adult Mice. Cell Metab, 26(3), 539-546.e535. doi:10.1016/j.cmet.2017.08.005

Schymanski, E. L., Jeon, J., Gulde, R., Fenner, K., Ruff, M., Singer, H. P., & Hollender, J. (2014). Identifying Small Molecules via High Resolution Mass Spectrometry: Communicating Confidence. *Environmental Science & Technology*, 48(4), 2097-2098. doi: 10.1021/es5002105.

Wanichthanarak, K., Fan, S., Grapov, D., Barupal, D. K., & Fiehn, O. (2017). Metabox: A Toolbox for Metabolomic Data Analysis, Interpretation and Integrative Exploration. *PLoS One*, 12(1), e0171046. doi: 10.1371/journal.pone.0171046.

Wu, D., Yu, H. Q., Xiong, H. J., Zhang, Y. J., Lin, X. T., Zhang, J., . . . Xie, C. M. (2021). Elevated Sodium Pump α3 Subunit Expression Promotes Colorectal Liver Metastasis via the p53-PTEN/IGFBP3-AKT-mTOR Axis. Front Oncol, 11, 743824. doi:10.3389/fonc.2021.743824
